# Investigation into ligand selectivity and bias at the formyl peptide receptor family

**DOI:** 10.1016/j.jpet.2025.103764

**Published:** 2025-10-28

**Authors:** Christine E. Jack, Christina M. Thomson, Sergio Dall’Angelo, Dawn Thompson, James N. Hislop

**Affiliations:** School of Medicine, Medical Sciences and Nutrition, Institute of Medical Sciences, University of Aberdeen, Aberdeen, Scotland

**Keywords:** Formyl peptide receptor, Ligand bias, Arrestin, Signalling, Bioluminescence resonance energy transfer

## Abstract

Formyl peptide receptors (FPRs) mediate both proinflammatory and resolution phases of the inflammatory response involved in many disease states. Harnessing their potential for pharmaceutical development requires an accurate picture of their signaling and regulation to the many test compounds developed. This study compares distinct responses of mouse and human FPR subtypes to several ligands in an attempt to clarify the dual nature of FPR signaling. Here, we expressed human and mouse variants of FPR1 and FPR2 in HEK293 cells and assessed competition binding, bioluminescence resonance energy transfer assays to measure the interaction between receptors and either Arrestin 3 or mini-Gsi, internalization, and extracellular signal-regulated kinase 1/2 phosphorylation. Concentration-response curves for 11 distinct ligands at each subtype were generated, then analyzed to determine EC50s, Emax values, and ligand bias. All compounds were less potent than WKYMVm across receptor subtypes, with the strength of signaling correlating with affinity estimates. The rank order of potency was maintained across the signaling pathways. Notably, MMK1 was specific for human FPR2, and BMS-986235 was selective for FPR2 over FPR1 in both species. Little evidence of pathway bias was detectable, with the notable exception of the recently described pepducin F2Pal10. The majority of tested ligands exhibit efficacy at each subtype, meaning conclusions of physiological receptor function based on these compounds should be treated circumspectly. It is not possible to determine distinct signaling profiles that would explain proresolution versus inflammatory physiology, and the most likely explanation for these data would be a combination of FPR1 and FPR2 responses.

**Significance Statement:**

No evidence of ligand bias between G-protein activation, arrestin recruitment, or internalization was found at formyl peptide receptors for 11 distinct agonists. Differences in physiological outcome are more likely to reflect efficacy at both subtypes rather than inherent signaling bias.

## 1 Introduction

Formyl peptide receptors (FPRs) are a subfamily of G-protein-coupled receptors (GPCRs) that play a vital role in the innate immune system for defense against injury and infection and thus represent an important target for therapeutic intervention for dysregulated inflammatory disease states.^1^, ^2^, ^3^ This interest is driven by the proposed functions that FPRs play in coordinating the inflammatory response, particularly the unique role of the FPR2 in promoting the resolution of inflammation.^3^^,^^4^ It is essential that following an inflammatory insult, resolution occurs promptly, allowing a return to homeostasis, as failure to do so leads to nonresolving inflammation, which is now recognized to be a contributing factor to several chronic diseases.^5^^,^^6^ The recent realization that chronic diseases are often driven by low-level inflammation that fails to resolve and that resolution is an active process requiring initiation has highlighted the importance of FPR2 in therapeutic development (reviewed in^1^^,^^2^^,^^7^).

One of the striking features of the FPR2 is the number and variety of structurally diverse ligands proposed to act at this receptor yet are able to promote completely opposing effects on the inflammatory response (see reviews^3^^,^^8^). Deciphering the pharmacological basis behind this is critical for understanding the regulation of the inflammatory response while providing a novel approach in the treatment of chronic inflammatory diseases. Since their cloning and isolation in 1992^9^^,^^10^ there have been numerous ligands identified that reportedly activate FPR2 to induce either proinflammatory or proresolving responses.^4^^,^^7^ However, due to the numerous cell and tissue backgrounds, assay readouts, lack of receptor-specific ligands, and reference ligands in the determination of these potencies and functions, the true nature of many of these FPR2 ligands is unclear.

One source of variation is that much of the data are generated using either whole animal mouse models, primary immune cells isolated from mice, or immune cells isolated from human blood with the assumption that the pharmacology is interchangeable, with a relative paucity of research directly comparing the pharmacology and functions of the human and murine FPR counterparts. While human FPR1-3 and mouse FPR1-3 do share high levels of structural homology and cellular expression, the murine FPR family is expanded relative to the human FPRs, and thus the ligand recognition and response invoked by the murine FPRs (and mice in general) may be very different.^11^^,^^12^ Despite the clear importance of the FPR family, the few comparative studies conducted so far have focused on comparing within the human FPRs^11^^,^^13^, ^14^, ^15^, ^16^ or within the murine Fprs^17^ with even fewer comparing equivalent receptors across species.^18^, ^19^, ^20^, ^21^ As these many comparative studies are conducted in different cellular and tissue backgrounds without inclusion of reference ligands, definitive conclusions across studies are difficult to make regarding the efficacy and selectivity of many of the proposed ligands for human and mouse FPR2. Thus, a greater understanding of mouse FPR pharmacology alongside human FPRs is required if murine models are to aid in the discovery and development of proresolution therapies targeting FPR2 and not be limited in their translatability between species.

In recent years, the phenomenon of ligand-directed signaling, or biased signaling, has become predominant in GPCR research, where distinct ligands can have distinct signaling responses through the same receptor.^22^^,^^23^ This can be observed as a preference for activation of a specific G-protein over another (eg, Gi vs Gq) or via distinct functional responses. More typically, bias is reported as a preference for activation of G-protein over an interaction with arrestin or vice versa.^23^ At the physiological level, the existence of both proinflammatory and proresolution ligands activating the FPR2 can be thought of as a type of ligand bias^24^ and it is not unreasonable to suggest that ligand bias might underlie the reported differences in physiological effects observed with different ligands at this receptor. Indeed, recent studies have tried to compare FPR ligands at FPR2,^25^ or between human FPR1 and FPR2^26^^,^^27^; however, none of these compared agonist profiles across human and mouse species and for both FPR1 and FPR2.

Here, we address these issues by a direct comparison of human and mouse FPR pharmacology in a single heterologous expression system. Human FPR1 (Hs_FPR1), human FPR2 (Hs_FPR2), mouse Fpr1 (Mm_Fpr1), and mouse Fpr2 (Mm_Fpr2) were each expressed in HEK293 cells to allow unambiguous interpretation for each individual receptor. We tested several reported FPR ligands with diverse structures from different origins, that is, bacterial/cellular-derived peptides (fMLP, fMLFK, MCT-2), small molecules (QuinC1, TCFPR43, BMS-986235, and ACT-389949), and synthetic peptides (WKYMVM, MMK-1, and F2Pal10), with each ligand response being compared to a reference ligand, the synthetic peptide WKYMVm.^28^^,^^29^ This straightforward approach allows for a direct comparison of ligand specificities and efficacies between the human and mouse FPR subtypes within the same cellular background, allowing for a clearer interpretation of the pharmacology of these reported ligands that will prove to be invaluable to the understanding of the effects of these ligands in immunophysiology.

## 2 Materials and methods

### 2.1 Cell culture

HEK293 cells (RRID:CVCL_0045) were cultured in Dulbecco’s modified Eagle’s medium supplemented with 10% FBS and routinely passaged by washing and lifting in PBS with EDTA (Lonza). Transfections were carried out by using linear polyethylenimine hydrochloride (MW 40,000, Polysciences Inc). DNA was incubated in 150 mM sterile NaCl and then mixed with 1 mg/ml polyethylenimine hydrochloride at a ratio of 5 *μ*l for each *μ*g of DNA. Stable cell lines were generated by culturing cells at low confluency with 100 *μ*g/ml zeocin for 3 weeks. Resistant colonies were then screened for expression of the FLAG epitope by immunofluorescent microscopy.

### 2.2 Constructs and reagents

The cDNA for N-terminal FLAG-tagged human FPR2 (Hs_FPR2) has already been described,^30^ cDNA of the Hs_FPR1 and the mouse orthologs Mm_Fpr1 and Mm_Fpr2 were purchased from cDNA.org, and ThermoFisher, respectively. Receptors were N-terminal FLAG-epitope tagged by the use of the HiFi assembly protocol from New England Biolabs. Briefly, cDNA was amplified by polymerase chain reaction using primers with homology to the SS-FLAG vector previously described,^30^ and recombined with BamHI-digested vector according to the manufacturer’s instructions. For bioluminescence resonance energy transfer (BRET)-based assays, the SS-FLAG-tagged receptors were then subcloned into pLuc8-N1 (a generous gift from N. Lambert^31^), again using the HiFi assembly protocol. Yellow fluorescent protein-tagged Arrestin 3 and Venus-tagged miniGsi BRET constructs have been previously described^31^^,^^32^ and were generously provided by Meritxell Canals (University of Nottingham) and Nevin Lambert (Augusta University). The M1-antiflag antibody was purchased from Sigma and then conjugated with Alexa647 using the antibody labeling kit (ThermoFisher) according to the manufacturer’s instructions. WKYMVm, WKYMVM, MMK1, QuinC1, TC-FPR43, and fMLP were purchased from Tocris; BMS-986235 and ACT-389949 were purchased from MedChemExpress UK; and fMet-Leu-Phe-Lys (fMLFK) was purchased from Cambridge Biosciences. All compounds were dissolved in DMSO apart from WKYMVm/M, which was soluble in water. Vinculin (E1E9V), *β*-tubulin (D3U1W), and phospho-p44/42 extracellular signal-regulated kinase (ERK) 1/2 (Thr202/Tyr204) (D13.14.4E) XP antibodies were all purchased from cell signaling technology.

### 2.3 Synthesis of F2Pal10, MCT-2, and fluorescent WKYMVm

All reagents used were of analytical, peptide synthesis, or high-performance liquid chromatography grade. 9-Fluorenylmethoxycarbonyl (Fmoc)-protected amino acid monomers; Oxyma Pure, and Fmoc-preloaded-Wang resins were obtained from CEM Corporation. Trifluoroacetic acid, *N*,*N*’-diisopropylcarbodiimide, Formyl-l-Methionine (For-Met-OH), Fmoc-d-Methionine-OH, and palmitic acid were obtained from Fluorochem. Fmoc-Lys(5/6-FAM)-OH was obtained from Stratech UK. *N,N’*-dimethylformamide (DMF); dichloromethane; diethyl ether; and high-pressure liquid chromatography (HPLC)-grade acetonitrile and water were obtained from VWR Chemicals. Piperidine, 2,2′-(ethylenedioxy)diethanethiol, and 4-methylbenzhydrylamine Rink Amide high load and low load resins were obtained from Merck/Sigma Aldrich.

Peptides were synthesized exploiting Fmoc (*N*-(9-fluorenyl) methoxycarbonyl) solid-phase microwave-assisted peptide chemistry using a Liberty Blue™ Automated Microwave Peptide Synthesizer (CEM).^33^ Coupling steps were performed using the Carbomax strategy^34^: 1 M *N,N'*-diisopropylcarbodiimide solution in DMF as a coupling agent and a 1 M Oxyma pure (ethyl cyano(hydroxyimino)acetate) in DMF solution as additive. Fmoc deprotection was performed using a solution of 20% piperidine in DMF.

For the synthesis of terminally formylated peptide MCT-2 (Formyl-MTPMRKINPLMKLIN from Cytochrome b^35^) and the peptide bearing the fluorescein dye (K(5/6-FAM)-WKYMVm-NH_2_), the software was edited to include 2 new amino acids (For-Methionine and Fmoc-Lys(FAM)-OH), and the synthesis was performed according to the standard procedure described above. For the synthesis of the F2Pal10 (Pal-Pal-KIHKKGMIKS) peptide, the software was edited to include palmitic acid, and the synthesis was performed using the standard procedure as described above. N-terminal palmitoylation was performed on the resin as the last step of the synthesis.

Cleavage of the synthesized peptide from the resin and removal of side protecting groups was performed by treating the resin with a cleavage solution composed of 92.5% trifluoroacetic acid, 2.5% TIS, 2.5% 2,2′-(ethylenedioxy)diethanethiol, and 2.5% water for 3 hours at room temperature. Trifluoroacetic acid was removed by a stream of nitrogen, and the peptide was precipitated by the addition of cold diethyl ether. Crude peptides were purified by preparative RP-HPLC using an Agilent 1260 system and a Phenomenex Luna C18(2) preparative column (5 *μ*m, 100 Å, 21 mm × 250 mm I.D. × L) using an optimized gradient.

Purity of the peptides was evaluated by HPLC-MS analysis using an Agilent 1200 HPLC equipped with a diode array detector and coupled with a single quadrupole mass detector using a Phenomenex Luna C18 analytical column (5 *μ*m, 100 Å, 4.6 mm × 250 mm I.D. × L) and the appropriate eluent gradient (see Supporting Info for specific information).

### 2.4 Competition binding of fluorescent WKYMVm

Binding of fluorescent WKYMVm was performed by adapting a standard radioligand binding assay.^36^ Briefly, HEK293 cells stably expressing the different receptors were lifted in PBS and incubated on ice with 10 nM K(5/6-FAM)-WKYMVm and either PBS or increasing concentrations of unlabeled ligand for 90 minutes (to allow equilibrium binding). Mean cell fluorescence was then measured for 5000 cells by flow cytometry using either a BD FACScalibur or BD Attune. All data are expressed as a percentage of mean total fluorescence detected for WKYMVm binding with no competitor present.

### 2.5 Internalization of FLAG-tagged receptors by flow cytometry

Internalization of FPRs was measured by flow cytometry.^30^ Briefly, cells were incubated with Alexa Fluor 657 conjugated M1 antiflag antibody (Sigma-Aldrich Cat# F3040, RRID:AB_439712) for 30 minutes at 37 °C to label surface receptors. Cells were then incubated with the indicated concentration of ligand before washing in PBS/0.04% EDTA to remove any remaining surface antibody. Remaining fluorescence was then measured using flow cytometry on either a BD FACScalibur or Attune, recording 5000 gated events. Data were then expressed as the mean fluorescence detected as a percentage of that seen with 1 *μ*M WKYMVm, which was used as a reference compound throughout. As a control, some samples were left untreated, and “strip only” was measured as a readout of constitutive endocytosis, and this value was subtracted from the data. Vehicle control (0.1% DMSO) had no effect on internalization of receptors.

### 2.6 BRET measurements of receptor activation

BRET1 assays to measure FPR interaction with Venus-mGsi and Arrestin 3-yellow fluorescent protein (YFP) were performed as previously described.^31^^,^^32^ Briefly, all FPRs C-terminally fused to Renilla Luciferase (donor, RLuc8) were transiently transfected along with either Venus-mGsi or YFP-Arrestin 3 (acceptor) at a ratio of 1:4. 24 hours post transfection, cells were replated into white-walled, 96-well plates and cultured for a further 24–48 hours. On the day of experimentation, growth media was aspirated and replaced with 80 *μ*l Hanks Buffered Saline Solution and equilibrated for 30 minutes. Coelenterazine *h* was added to each well to a final concentration of 5 *μ*M for 5 minutes before the addition of each compound, and the plate was incubated for a further 10 minutes at 37 °C before being read on a ClarioStar Plus, with luminescence measured at 480/20 nm (donor) and 530/20 (acceptor). The BRET ratio (acceptor/donor) was determined, and the background (untreated) subtracted to give the N-BRET value. This was then normalized to the N-BRET value observed for 1 *μ*M WKYMVm, which was measured on each plate.

### 2.7 Measurement of pERK by Western blotting

ERK phosphorylation was detected by Western blotting as previously described.^30^ Briefly, HEK293 cells stably expressing FLAG-tagged FPRs were plated into 12-well culture plates until 90% confluent. On the day of the experiment, culture media was replaced with serum-free Dulbecco’s modified Eagle’s medium and incubated for 2 hours before stimulation with the indicated compounds. After 7 minutes, the media was removed and stimulation terminated by the addition of 100 *μ*l sample buffer (0.5% SDS, Tris (pH 6.8), glycerol, at >90 °C). Samples were then separated by SDS-PAGE and transferred to nitrocellulose membranes before incubating overnight at 4 °C with phospho-ERK antibody (Cell Signaling Technology Cat# 4370, RRID:AB_2315112). Blots were stripped and reprobed with either vinculin (Cell Signaling Technology Cat# 4650, RRID:AB_10559207) or *β*-tubulin antibodies to ensure equal loading. Each blot contained 1 lane corresponding to lysate from cells treated with 1 *μ*M WKYMVm from the same experiment, which was used for normalization with data expressed as a percentage of the phospho-ERK intensity.

### 2.8 Data analysis

To allow for any experimental variation in transfection efficiency, Western blot transfer and detection, or M1 antibody labeling intensity, all studies included a control stimulation (1 mM WKYMVm), against which all responses were normalized. All data were analyzed using GraphPad Prism using the nonlinear regression log(agonist) versus response (3 parameters) function for all assays, except binding, which was analyzed using the One site-FitlogIC50 function. Bias calculations were carried out following IUPHAR guidelines as described,^27^^,^^37^ and data were presented as the Δ Activity ratio (Log[Emax_B_/EC50_B_])-(Log[Emax_A_/EC50_A_]).

## 3 Results

### 3.1 Binding of compounds to the different FPRs

**Fig. 1:**
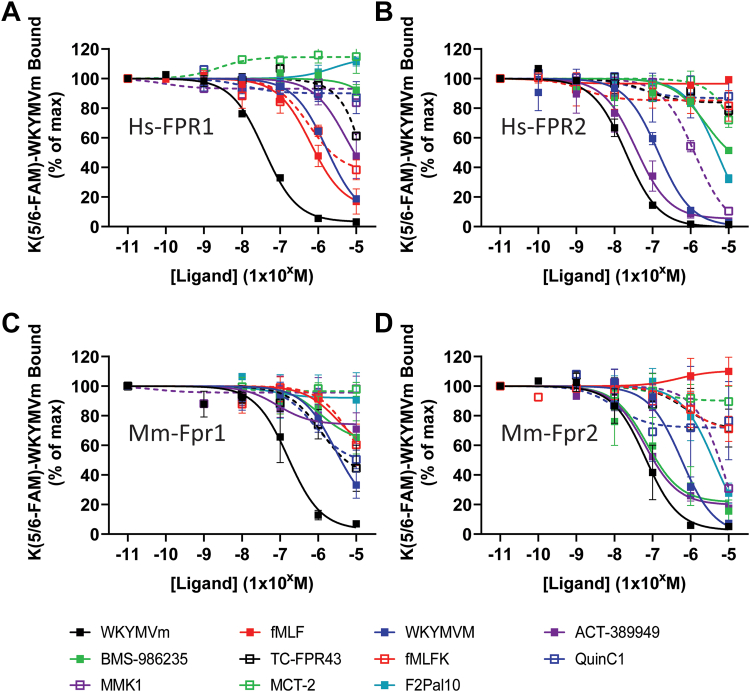
Binding of different ligands to FPR. HEK293 cells stably expressing FLAG-tagged Hs_FPR1 (A), Hs_FPR2 (B), Mm_Fpr1 (C), or Mm_Fpr2 (D) were incubated on ice with 10 nM K(5/6-FAM)-WKYMVm in the presence of the indicated compound before analysis of mean fluorescence by flow cytometry. Data shown are the mean +/− SEM expressed as a percentage of the fluorescence obtained in the presence of no additional ligand (*n* = 3–5).

All ligands tested have been previously described as agonists at 1 or more of the FPRs.^8^^,^^28^^,^^29^ As a starting point, we generated HEK293 cells stably expressing each of the 4 FPRs investigated, which were then used for subsequent assays. To delineate the relative specificity for each ligand, we developed a ligand binding assay based on traditional radioligand competition binding,^36^ using fluorescein-labeled WKYMVm (K(5/6-FAM)-WKYMVm, Fig. 1; Supplemental Fig. 1 and Table 1). Initial studies revealed that this compound showed saturable binding with a Kd in the low nM range at hFPR2 (not shown), and using 10 nM allowed a sufficient dynamic range for competition analysis at each of the 4 receptors investigated. Competition binding was then investigated for each ligand at each of the receptors. It was immediately recognized that none of the ligands tested showed higher affinity for any of the receptors than WKYMVm, and many of the compounds showed little or incomplete competition with K(5/6-FAM)-WKYMVm at all receptors. Indeed, at Mm_fpr1, no compound tested was able to fully compete with 10 nM K(5/6-FAM)-WKYMVm, even at concentrations of 10 *μ*M (Fig. 1C). Similarly, at Hs_FPR1, only the enantiomers WKYMVM and fMet-Leu-Phe (fMLF) were able to fully compete K(5/6-FAM)-WKYMVm, although incomplete competition was also observed for fMLFK, ACT-389949, and TC-FPR43, indicating these ligands have affinity for the orthosteric binding pocket of Hs_FPR1 (Fig. 1A). In contrast (Fig. 1B), ACT-389949, WKYMVm, and MMK1 produced complete displacement of K(5/6-FAM)-WKYMVm at the Hs_FPR2 (WKYMVm > ACT-389949 > WKYMVM > MMK1). Similar data were observed for Mm_fpr2 (Fig. 1D), but noticeably MMK1 showed little displacement in comparison to Hs_FPR2. Interestingly, the pepducin F2Pal10 was able to displace K(5/6-FAM)-WKYMVm at higher concentrations at both Hs_FPR2 and Mm_Fpr2, even though its mechanism of action is thought to be intracellular.^38^ Finally, BMS-986235 showed incomplete competition (∼50%) at Hs_FPR2, even at 10 *μ*M; however, a higher relative affinity was observed at Mm_Fpr2, although this may reflect a difference in affinity of WKYMVm between the different receptor species.

**Table 1:** The different IC50 and maximum proportion of competition with 10 nM WKYMVm at each receptor following incubation with each ligand

Binding			WKYMVm	WKYMVM	ACT-389949	BMS-986235	fMLF	fMLFK	TC-FPR43	QuinC1	MCT-2	MMK1	F2Pal10
Hs-FPR1	Log IC50 (M)	Mean	−7.419	−5.778	−5.265	ND	−6.194	−6.232	ND	−7.943	ND	−10.05	ND
		SEM	1.587	0.069	0.531	ND	0.114	0.229	ND	0.865	ND	2.269	ND
		*P* value	-	.0001	.073	-	.0007	.029	-	.864	-	.372	-
	Max Comp (%)	Mean	96.85	94.87	80.70	ND	88.01	63.28	ND	9.96	ND	6.82	ND
		SEM	0.043	4.325	39	ND	5.533	7.851	ND	3.205	ND	2.081	ND
		*P* value	-	.964	.741	-	.276	.03	-	<.0001	-	<.0001	-
		*N*	3	3	4	3	5	4	3	4	3	4	3
Hs_FPR2	Log IC50 (M)	Mean	−7.702	−6.847	−7.434	−5.612	−9.585	−8.833	−7.174	−7.399	ND	−5.88	−5.27
		SEM	0.0513	0.065	0.153	0.272	6.431	0.975	0.464	1.235	ND	0.051	0.14
		*P* value	-	.0006	.506	.005	.784	.429	.533	.864	-	<.0001	.0002
	Max Comp (%)	Mean	100	99.83	94.77	60.14	3.4	14.72	16.25	13.23	ND	100	100
		SEM	1.898	2.947	5.533	11.53	1.179	4.046	3.019	6.298	ND	3.337	13.16
		*P* value	-	.966	.722	.053	<.0001	<.0001	<.0001	<.0001	-	>.999	>.999
		*N*	3	4	4	3	3	4	3	3	3	3	3
Mm_Fpr1	Log IC50 (M)	Mean	−6.787	−5.554	−7.070	−5.951	−5.498	−5.083	−5.926	−6.114	ND	ND	ND
		SEM	0.157	0.249	0.532	0.324	0.406	0.986	0.324	0.219	ND	ND	ND
		*P* value	-	.0157	.896	.324	.114	.429	.239	.27	-	-	-
	Max Comp (%)	Mean	97.05	85.43	25.96	37.54	45.65	72.44	61.39	52.13	ND	ND	ND
		SEM	6.984	15.44	5.643	10.21	13.91	78.40	12.52	6.588	ND	ND	ND
		*P* value	-	.951	.002	.02	.072	.822	.075	.005	-	-	-
		*N*	3	3	4	4	5	5	4	5	5	4	2
Mm_Fpr2	Log IC50 (M)	Mean	−7.184	−6.233	−7.192	−7.139	ND	−6.082	−6.16	−7.98	−6.883	ND	−5.377
		SEM	0.161	0.137	0.532	0.305	ND	0.477	0.828	1.109	1.6–67	ND	0.373
		*P* value	-	.0157	.969	.900	-	.156	.533	.823	.863	-	.004
	Max Comp (%)	Mean	97.54	100	80.31	78.55	ND	30.81	29.56	27.99	9.97	ND	100
		SEM	6.554	7.431	2.421	10.15	ND	8.623	13.16	11.64	7.524	ND	31.51
		*P* value	-	.966	.232	.167	-	.004	.008	.005	.0001	-	.995
		*N*	4	3	3	4	3	3	5	3	4	3	3

*P* values were obtained using multiple *t* tests and indicate a difference from WKYMVm (ND denotes no competition observed).

### 3.2 Formyl peptide receptor interaction with mGsi following activation

**Fig. 2:**
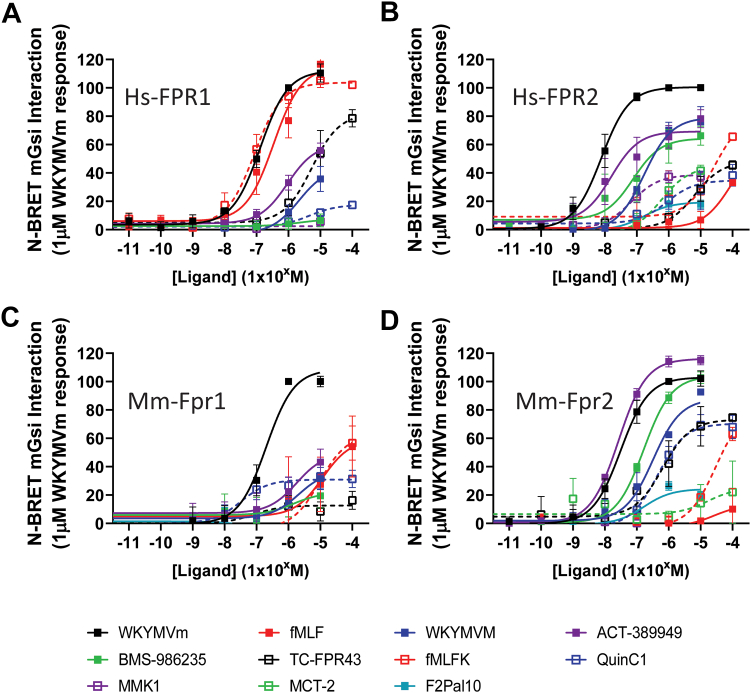
Interaction of FPR with mGsi induced by different ligands. HEK293 cells were transiently transfected with C-terminally RLuc8-tagged Hs_FPR1 (A), Hs_FPR2 (B), Mm_Fpr1 (C), or Mm_Fpr2 (D) and Venus-mGsi before replating into white, 96-well plates. Cells were incubated with 5 mM coelenterazine *h* for 5 minutes and the indicated ligands for 10 minutes before reading. Data shown are the mean +/− SEM normalized BRET ratio expressed as a percentage of that seen with 1 mM WKYMVm for each receptor (*n* = 3–6).

Although most of the compounds tested showed little competition with K(5/6-FAM)-WKYMVm, this likely reflects the relatively high affinity that WKYMVm has for FPRs, rather than the other compounds completely lacking affinity. We therefore decided to screen each compound for efficacy at causing a receptor-G-protein interaction as measured by BRET.^31^ Preliminary studies using Hs_FPR2 revealed that only the mGsi construct showed an agonist-induced increase in association (Supplemental Methods, Supplemental Fig. 2), and therefore used this for construct for the comparison study herein. We used the mini-Gsi-Venus construct, which is based on Gs but has the C-terminal domain of Gi, lacking the beta-gamma interaction domain and therefore unable to hydrolyze GTP following nucleotide exchange, leading to a long-lasting interaction following receptor activation.^31^ We created C-terminal fusion proteins of the FLAG-FPR fused in frame with the *Renilla* Luciferase 8 (F-FPR-Luc) and then the BRET assay to systematically investigate each ligand with each of the FPRs (Fig. 2; Supplemental Fig. 3 and Table 2).

**Table 2:** The different EC50 and Emax observed for interaction with G-protein at each receptor following incubation with each ligand

mGsi			WKYMVm	WKYMVM	ACT-389949	BMS-986235	fMLF	fMLFK	TC-FPR43	QuinC1	MCT-2	MMK1	F2Pal10
Hs-FPR1	Log EC50 (M)	Mean	−6.88	−5.65	−6.47	−5.65	−6.47	−7.07	−5.27	−5.34	ND	ND	ND
		SEM	0.074	0.297	0.157	2.357	0.188	0.118	0.252	0.475	ND	ND	ND
		*P* value	-	.038	.032	.82	.132	.256	.011	.065	-	-	-
	Emax (%)	Mean	112.10	44.06	60.56	7.40	114.65	103.70	81.94	17.60	ND	ND	ND
		SEM	3.42	9.13	4.97	7.36	9.27	4.47	9.43	3.89	ND	ND	ND
		*P* value	-	.005	.004	.0008	.83	.398	.04	.0002	-	-	-
		*N*	3	4	3	3	4	3	3	3	2	4	3
Hs_FPR2	Log EC50 (M)	Mean	−8.11	−6.77	−7.80	−7.12	−4.05	−4.57	−5.19	−6.06	−6.23	−7.17	−6.78
		SEM	0.093	0.126	0.279	0.343	0.733	0.489	0.227	0.613	0.308	0.196	0.388
		*P* value	-	.0013	.488	.068	.005	.001	<.0001	.048	.003	.01	.061
	Emax (%)	Mean	100.48	79.19	69.25	64.37	61.66	80.66	48.16	34.75	44.42	38.73	19.36
		SEM	3.19	4.30	5.92	7.87	52.28	22.52	5.41	6.67	6.91	2.63	3.03
		*P* value	-	.019	.012	.01	.66	.398	.0005	.0002	.001	<.0001	<.0001
		*N*	4	3	3	3	3	3	4	4	3	4	5
Mm_Fpr1	Log EC50 (M)	Mean	−6.69	−5.68	−5.74	−5.85	−4.93	−5.02	−7.09	−7.49	−7.78	ND	ND
		SEM	0.139	0.452	0.563	1.671	0.521	0.328	3.711	0.536	1.398	ND	ND
		*P* value	-	.115	.307	.82	.034	.007	.934	.275	.286	-	-
	Emax (%)	Mean	108.57	37.52	49.76	21.68	59.28	62.90	12.62	30.89	−8.19	ND	ND
		SEM	6.56	9.76	15.10	15.81	15.77	11.73	4.50	3.73	4.66	ND	ND
		*P* value	-	.003	.021	.007	.081	.063	<.0001	<.0001	.001	-	-
		*N*	4	6	3	3	4	5	6	6	2	5	4
Mm_Fpr2	Log EC50 (M)	Mean	−7.51	−6.49	−7.57	−6.78	−4.58	−4.52	−6.19	−6.35	−4.77	ND	−6.78
		SEM	0.10	0.17	0.04	0.05	0.67	0.20	0.30	0.15	1.54	ND	0.278
		*P* value	-	.0075	.558	.002	.011	<.0001	.012	.003	.168	-	.074
	Emax (%)	Mean	102.95	87.88	116.06	103.89	13.23	82.70	73.16	70.36	24.97	ND	24.24
		SEM	3.88	8.45	1.74	2.08	7.89	11.85	7.97	3.94	20.47	ND	2.81
		*P* value	-	.133	.022	.83	.0004	.398	.03	.001	.007	-	<.0001
		*N*	4	3	4	4	3	4	4	4	3	3	3

*P* values were obtained using multiple *t* tests and indicate a difference from WKYMVm (ND denotes no interaction detected).

For all receptors we used WKYMVm as a reference ligand, as this shows a clear, sigmoidal, concentration-dependent increase in BRET ratio across all FPRs (Supplemental Fig. 3A), and subsequently normalized all data to the response observed for 1 *μ*M. For Mm_fpr1, WKYMVm was in fact the only ligand tested that showed “full” efficacy, with all other compounds being either partial agonists (eg, QuinC1 and TC-FPR43) or weak agonists that had not yet achieved Emax, with BRET signals only detected at 1 *μ*M or greater (fMLP, fMLFK, ACT-389949). In contrast, the formylated peptides fMLF and fMLFK were equally potent as WKYMVm at the Hs_FPR1, despite having little efficacy at Mm_fpr1.^17^ Interestingly, the FPR2 “selective” ligands WKYMVM and ACT-389949 both induced a clear interaction with mGsi at both FPR1 species, albeit at concentrations of 1 mM and above, whereas no detectable signal was observed for BMS-986235, MMK1, or MCT-2 (Fig. 2A).

We next examined the human and mouse FPR2 receptors, and the rank order of potency observed was broadly the same at both receptor species (Fig. 2, B and D). Of interest, however, is that ACT-389949 and BMS-986235 were partial agonists at Hs_FPR2, and MMK1 was only an agonist (partial) at Hs_FPR2 with no effect observed at Mm_Fpr2. Of further note is that fMLFK, which is sometimes reported as a proinflammatory ligand at FPR2, only shows agonistic activity at 10 *μ*M or above, far greater concentrations than required to activate Hs_FPR1 or even Mm_fpr1. Finally, the pepducin, F2Pal10, which has been shown to cause activation of the FPR2 receptors,^38^^,^^39^ was unable to induce a strong interaction of mGsi with either Hs_FPR2 or Mm_Fpr2. This result was somewhat surprising, as F2Pal10 has been previously reported to strongly activate signaling through FPR2 using physiological responses, although how this is achieved is unknown. We therefore used the TruPath system (Supplemental Methods^40^) to determine if F2Pal10 is able to initiate Gi dissociation. Unsurprisingly, WKYMVm exhibited a potent and robust dissociation of G*α*i2 from *β*3*γ*9, whereas F2Pal10, although noticeably less potent than WKYMVm (EC50 of 130 nM compared to 0.9 nM), was able to elicit a maximal response, in stark contrast to the observed partial mGsi interaction (Supplemental Fig. 4).

### 3.3 Activation of signaling cascades downstream of FPRs

**Fig. 3:**
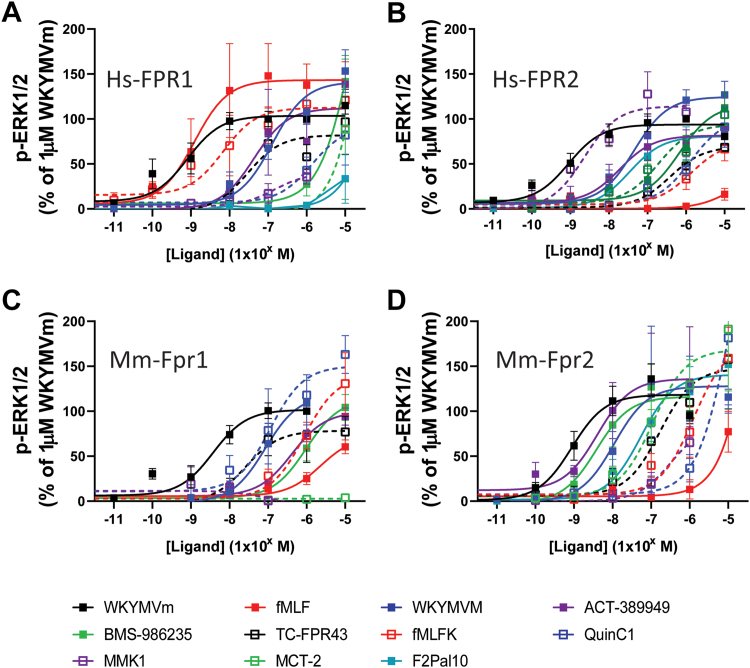
ERK phosphorylation by different ligands at the FPR. HEK293 cells stably expressing FLAG-tagged Hs_FPR1 (A), Hs_FPR2 (B), Mm_Fpr1 (C), or Mm_Fpr2 (D) were stimulated with indicated ligands for 10 minutes before lysis and analysis by SDS-PAGE and Western blotting. Data shown are the mean +/− SEM expressed as a percentage of the intensity observed to 1 mM WKYMVm for each receptor (*n* = 3–6).

Although a relatively simple and reproducible assay, interaction between the G-protein and the receptor is not necessarily an indication of activation of the G-protein and the initiation of signal transduction.^41^ Further, although we found no evidence of interaction with other G-proteins, previous studies have suggested that FPR2 might additionally signal through Gs and Gq.^26^^,^^27^ Therefore, to determine the efficacy of each compound in driving intracellular signaling, we investigated each ligand’s capability to induce phosphorylation of ERK1/2 (Fig. 3; Supplemental Fig. 5 and Table 3). Although ERK phosphorylation by FPRs is reported to be wholly Gi mediated.^42^, ^43^, ^44^ It is a robust readout that can be useful in detecting GPCR activation regardless of G-protein coupling (G_s_, G_i/o_, G_q/11_, and G_12/13_) as well as being reportedly activated by G-protein-independent processes,^45^^,^^46^ and as such, ensures that any highly biased ligands (eg, do not initiate Gi interaction) are not missed. Stable cell lines expressing the different receptors were first stimulated with various concentrations of our reference ligand WKYMVm before lysis and detection by Western blotting. Clear concentration-response curves were observed with WKYMVm for each receptor tested with EC50 values in the low nM range and a rank order of potency of Mm_Fpr2 = Hs_FPR2 = Hs_FPR1 ≥ Mm_Fpr1 (Fig. 3A, black closed symbols; Supplemental Fig. 5A and Table 3). As with previous assays, 1 *μ*M WKYMVm was determined to be sufficient to evoke a maximum response at all receptors and was used as a normalization value for all subsequent studies. All ligands tested showed some efficacy at hFPR2, with the exception of fMLF, where pERK1/2 was only minimally detected following stimulation at 10 *μ*M (Fig. 3B). The related formylated peptide fMLFK was also only a weak agonist, but in common with previous findings, this longer peptide did elicit a greater response. The recently described formylated mitochondrial cryptic peptide, MCT-2,^35^^,^^47^^,^^48^ showed greater agonism than the bacterially derived compounds. Of the synthetic peptides tested, both the L-enantiomer WKYMVm and the peptide agonist MMK1 showed full agonism, albeit with a potency 50->100 less than WKYMVm. Unlike in the mGsi recruitment assay, both ACT-389949 and BMS-986235 were both full agonists, with ACT-389949 >200-fold more potent than BMS-986235 (compared to only 20-fold in the Gsi recruitment). Finally, the pepducin agonist F2Pal10^38^^,^^39^ was indeed shown to behave as an agonist, in contrast to its relatively low efficacy at recruiting mGsi. Overall, a similar trend was seen at the Mm_Fpr2 as for Hs_FPR2 (Fig. 3D), with the only difference again being that of MMK1, which shows efficacy only at 1 *μ*M or greater.

**Table 3:** The different EC50 and Emax observed for phosphorylation of ERK1/2 at each receptor following incubation with each ligand

P-ERK			WKYMVm	WKYMVM	ACT-389949	BMS-986235	fMLF	fMLFK	TC-FPR43	QuinC1	MCT-2	MMK1	F2Pal10
Hs-FPR1	Log EC50 (M)	Mean	−9.1	−6.887	−7.37	−4.79	−8.915	−8.164	−7.53	−5.86	ND	−6.84	ND
		SEM	0.200	0.214	0.511	1.10	0.405	0.453	0.468	0.524	ND	0.415	ND
		*P* value	-	.0007	.068	<.0001	.999	.381	.121	<.0001	-	.0027	-
	Emax (%)	Mean	103.5	141.3	112.1	ND	143.2	112.4	81.97	92.57	ND	40.15	ND
		SEM	5.46	11.99	19.11	ND	13.38	11.90	12.70	26.63	ND	9.52	ND
		*P* value	-	.999	.999	-	.999	.999	.999	.999	-	.999	-
		*N*	9	6	3	3	5	6	3	5	4	4	3
Hs_FPR2	Log EC50 (M)	Mean	−9.048	−7.347	−7.723	−6.168	−4.824	−5.881	−6.423	−5.882	−6.721	−8.646	−7.472
		SEM	0.173	0.147	0.299	0.275	1.385	0.33	0.232	0.281	0.223	0.21	0.238
		*P* value	-	.022	.381	.0002	<.0001	<.0001	.0009	<.0001	.0002	.997	.107
	Emax (%)	Mean	93.79	124.6	81.57	116.1	40.22	74.28	69.38	101.7	93.91	114.2	80.81
		SEM	4.583	7.095	7.46	15.58	76.74	13.65	7.098	16.25	8.48	7.95	6.68
		*P* value	-	.869	.998	-	.495	.978	.968	-	-	-	-
		*N*	11	6	3	4	5	6	4	4	8	4	4
Mm_Fpr1	Log EC50 (M)	Mean	−8.427	−7.077	−6.383	−6.008	−5.644	−6.070	−7.589	−6.945	ND	ND	ND
		SEM	0.182	0.351	0.191	0.198	0.269	0.316	0.487	0.383	ND	ND	ND
		*P* value	-	.089	.0005	<.0001	<.0001	.0002	.312	.0137	-	-	-
	Emax (%)	Mean	101.614	118.405	99.504	114.012	72.73	140.2	78.28	150.3	ND	ND	ND
		SEM	5.87	22.99	8.07	12.26	11.5	23.29	12.28	20.90	ND	ND	ND
		*P* value	-	.963	.999	.994	.639	.338	.879	.226	-	-	-
		*N*	6	5	4	4	7	7	4	4	1	4	-
Mm_Fpr2	Log EC50 (M)	Mean	−9.058	−7.91	−8.394	−8.422	ND	−5.907	−6.775	−4.94	−6.957	−6.482	−7.273
		SEM	0.221	0.378	0.596	0.300	ND	0.288	0.464	1.463	0.292	0.380	0.259
		*P* value	-	.802	.981	.986	-	.004	<.0001	<.0001	.065	.0278	.173
	Emax (%)	Mean	118.3	127.8	136.1	116.2	ND	176.1	147.8	381.2	168.7	86.59	140.8
		SEM	8.23	16.15	25.24	12.55	ND	28.75	27.58	678.3	21.16	24.04	13.69
		*P* value	-	.999	.999	.999	-	.999	.999	.971	.999	.999	.999
		*N*	7	3	4	4	3	4	4	5	4	4	5

*P* values were obtained using multiple *t* tests and indicate a difference from WKYMVm (ND denotes no pERK signal detected).

Similar to the mGsi assay, the majority of compounds tested had some efficacy at both Hs_FPR1 and Mm_Fpr1 (Fig. 3A). As expected, fMLF was as potent as WKYMVm at Hs_FPR1, with fMLFK less so, but still far greater than at Hs_FPR2. Importantly, we again observed clear agonist responses to ACT-389949 and WKYMVM, both of which are described as FPR2 selective. BMS-986235, MCT-2, MMK1, and F2Pal10 showed very little activity below 1 *μ*M. Finally, Mm_Fpr1 showed reduced potency to all ligands tested when compared to either the Hs_FPR1, Hs_FPR2, or Mm_Fpr2 (Fig. 3C).

### 3.4 Agonist-induced Arrestin 3 recruitment to FPRs

**Fig. 4:**
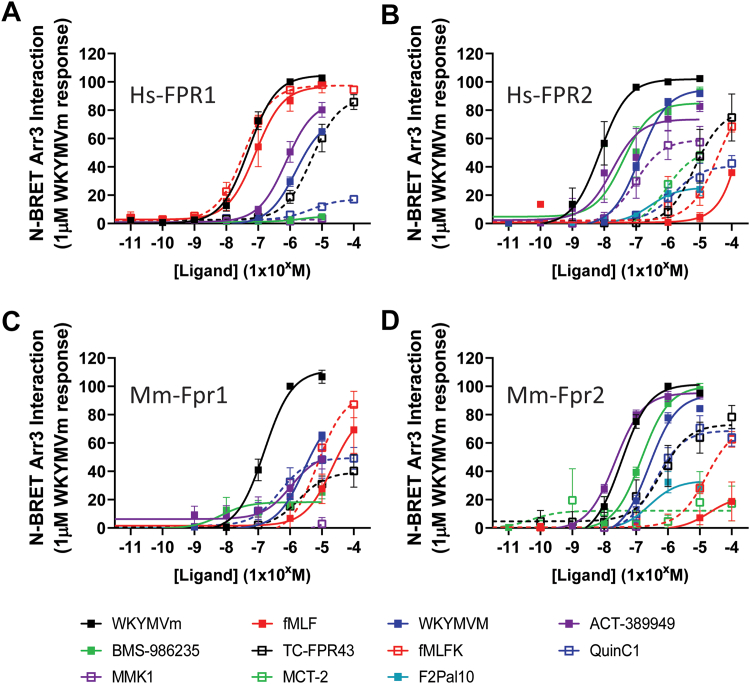
Interaction of FPR with Arrestin 3 induced by different ligands. HEK293 cells were transiently transfected with C-terminally RLuc8-tagged Hs_FPR1 (A), Hs_FPR2 (B), Mm_Fpr1 (C), or Mm_Fpr2 (D) and Arrestin 3-YFP before replating into white, 96-well plates. Cells were incubated with 5 mM coelenterazine *h* for 5 minutes and the indicated ligands for 10 minutes before reading. Data shown are the mean +/− SEM normalized BRET ratio expressed as a percentage of that seen with 1 mM WKYMVm for each receptor (*n* = 3–6).

Having thus far focused on G-protein activation, we next investigated the recruitment of arrestins to FPRs following receptor activation. For many GPCRs, the change in structure required to allow association and activation with G-alpha subunits can be distinct from those required to induce an interaction with arrestin, and this might underlie activation of different signaling cascades that can allow the identification of ligand bias.^49^ It is therefore conceivable that, although specific ligands may not cause an interaction with G-proteins, they might still promote an association with arrestins. We used the same BRET assay as with the mGsi but instead used Arrestin 3-YFP as the acceptor molecule (Fig. 4; Supplemental Fig. 6 and Table 4). In general, there were very few differences in ligand efficacies observed between the Arrestin 3 recruitment and those previously described for the recruitment of mGsi and p-ERK1/2 activation, with WKYMVm again being the most efficacious compound at all FPRs with slight differences in potency but the same rank order as for mGsi recruitment.

**Table 4:** The different EC50 and Emax observed for interaction with Arrestin 3 at each receptor following incubation with each ligand

Arrestin 3			WKYMVm	WKYMVM	ACT-389949	BMS-986235	fMLF	fMLFK	TC-FPR43	QuinC1	MCT-2	MMK1	F2Pal10
Hs-FPR1	Log EC50 (M)	Mean	−7.30	−5.82	−6.00	−5.64	−7.08	−7.45	−5.32	−5.35	ND	ND	ND
		SEM	0.06	0.07	0.10	1.28	0.13	0.08	0.14	0.22	ND	ND	ND
		*P* value	-	<.0001	.002	.267	.244	.200	.0006	.003	-	-	-
	Emax (%)	Mean	104.7	74.97	86.48	5.54	96.95	97.44	89.25	17.25	ND	ND	ND
		SEM	2.24	3.16	4.79	3.73	4.98	2.46	5.59	1.71	ND	ND	ND
		*P* value	-	.003	.048	<.0001	.274	.227	.06	<.0001	-	-	-
		*N*	3	4	3	3	4	3	3	3	3	4	3
Hs_FPR2	Log EC50 (M)	Mean	−8.13	−6.89	−7.74	−7.4	ND	−4.44	−5.16	−5.82	−6.06	−7.03	−6.55
		SEM	0.121	0.041	0.302	0.253	ND	0.374	0.326	0.344	0.173	0.303	0.475
		*P* value	-	.0008	.277	.115	-	.0002	.0006	.003	.0003	.029	.047
	Emax (%)	Mean	101.9	94.77	73.56	85.08	ND	92.71	80.35	40.71	51.77	59.09	25.51
		SEM	4.25	1.69	7.34	7.31	ND	24.72	13.71	5.36	5.13	7.02	5.03
		*P* value	-	.408	.046	.0178	-	.684	.227	.0003	.001	.002	<.0001
		*N*	4	3	4	4	3	3	4	4	3	4	5
Mm_Fpr1	Log EC50 (M)	Mean	−6.80	−5.56	−5.98	−8.17	−4.68	−5.14	−5.73	−6.33	ND	ND	ND
		SEM	0.067	0.099	0.331	0.733	0.246	0.159	0.384	0.384	ND	ND	ND
		*P* value	-	<.0001	.101	.152	.0006	.002	.033	.267	-	-	-
	Emax (%)	Mean	111.5	83.10	52.62	18.24	83.44	95.16	39.42	49.50	ND	ND	ND
		SEM	3.28	5.58	8.91	4.11	12.37	8.02	6.10	6.24	ND	ND	ND
		*P* value	-	.014	.004	<.0001	.148	.227	.0002	.0003	-	-	-
		*N*	4	5	3	3	3	4	4	4	3	4	5
Mm_Fpr2	Log EC50 (M)	Mean	−7.46	−6.59	−7.66	−6.82	−4.71	−4.79	−6.20	−6.40	−10.28	ND	−6.58
		SEM	0.075	0.083	0.051	0.045	0.593	0.149	0.271	0.162	3.35	ND	0.314
		*P* value	-	.0008	.142	.001	.01	<.0001	.008	.003	.361	-	.047
	Emax (%)	Mean	101.4	94.31	95.34	100.1	22.7	72.49	73.06	68.75	12.13	ND	33.42
		SEM	3.22	4.66	1.65	1.98	9.71	6.75	7.07	4.01	5.21	ND	4.54
		*P* value	-	.408	.144	.743	.0009	.033	.02	.001	<.0001		<.0001
		*N*	4	3	4	4	5	4	4	4	3	3	3

*P* values were obtained using multiple *t* tests and indicate a difference from WKYMVm (ND denotes no interaction detected).

As before, the bacterial formylated peptides fMLF and fMLFK were most efficacious at Hs_FPR1 by at least 100-fold compared to other receptor subtypes. However, fMLFK exhibited greater partial efficacy at Hs_FPR2, Mm_Fpr1, and Mm_Fpr2 at higher concentrations than that observed for fMLF. In comparison, ACT-389949 exhibited the highest potency of all compounds tested at Mm_fpr2; however, efficacy was also observed at all other subtypes. In contrast, BMS-986235 showed minimal ability to induce Arrestin 3 recruitment at either Hs_FPR1 or Mm_Fpr1 subtypes but does behave as a full agonist at both FPR2 species. Importantly, BMS-986235 was found to be as equipotent to ACT-389949 at Hs_FPR2. In addition, MMK-1 displayed partial agonism at Hs_FPR2 alone, maintaining the receptor subtype selectivity observed previously.

Surprisingly, the pepducin F2Pal10, which is reported to cause G-protein activation without arrestin recruitment, was observed to recruit Arrestin 3 to both Hs_FPR2 and Mm_Fpr2 (Fig. 4, C and D), with a similar potency, albeit weak, to that for mGsi (Fig. 2). Similarly, MCT-2 behaved as a weak partial agonist at both Hs_FPR2 and Mm_Fpr2 both mGsi recruitment and for Arrestin 3 recruitment. Likewise, the small molecule agonists, Quinc1 and TCFPR43, were partial agonists at all the receptor subtypes with similar rank orders of potency, Mm_Fpr2 > Hs_FPR1 > Hs_FPR2 > Mm_Fpr1.

### 3.5 Agonist-induced internalization of FPRs

**Fig. 5:**
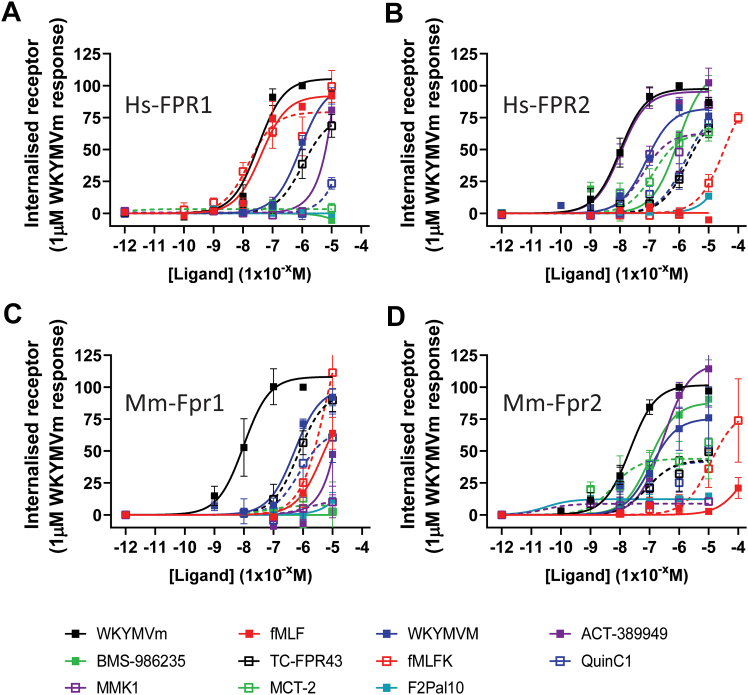
Internalization of FPR by different ligands. HEK293 cells stably expressing FLAG-tagged Hs_FPR1 (A), Hs_FPR2 (B), Mm_Fpr1 (C), or Mm_Fpr2 (D) were labeled with M1-AF647 antiflag antibody for 30 minutes before incubating for an additional 30 minutes with the indicated concentration of each ligand before analysis of mean internal fluorescence by flow cytometry. Data shown are the mean +/− SEM expressed as a percentage of the internalization to 1 mM WKYMVm for each receptor (*n* = 3–7).

Agonist-induced internalization is an important process in regulating cell signaling to prevent overstimulation and has been proposed to explain the “pharmacological tolerance” observed following chronic treatment with compounds in clinical development for FPR2.^24^^,^^50^ Therefore, because a final readout, we sought to determine whether each ligand was able to induce internalization of FPRs (Fig. 5; Supplemental Fig. 7 and Table 5), using a modified method of flow cytometry that exploits the calcium sensitivity of the FLAG-M1 antibody.^30^^,^^51^ Briefly, mature plasma membrane receptors were fluorescently labeled, either untreated or stimulated with an agonist, followed by a PBS-EDTA strip. Internalized receptors are protected from the “strip wash” and subsequently quantified and expressed as a percentage change from untreated.^30^

**Table 5:** The different EC50 and Emax observed for internalization of each receptor following incubation with each ligand for 30 minutes

Internal			WKYMVm	WKYMVM	ACT-389949	BMS-986235	fMLF	fMLFK	TC-FPR43	QuinC1	MCT-2	MMK1	F2Pal10
Hs-FPR1	Log EC50 (M)	Mean	−7.485	−6.059	ND	ND	−7.385	−7.79	−5.978	ND	ND	ND	ND
		SEM	0.121	0.074	ND	ND	0.131	0.232	0.181	ND	ND	ND	ND
		*P* value	-	.0016	-	-	.844	.372	.0046	-	-	-	-
	Emax (%)	Mean	105.5	100.9	ND	ND	92.10	79.30	76.10	ND	ND	ND	ND
		SEM	5.59	5.08	ND	ND	4.66	6.80	8.50	ND	ND	ND	ND
		*P* value	-	.715	-	-	.364	.147	.128	-	-	-	-
		*N*	3	3	4	4	3	5	3	4	3	3	3
Hs_FPR2	Log EC50 (M)	Mean	−8.004	−7.116	−7.975	−6.041	−8.946	−4.476	−5.729	−5.809	−6.869	−7.216	ND
		SEM	0.120	0.117	0.113	0.133	20.016	0.132	0.174	0.241	0.231	0.280	ND
		*P* value	-	.0009	.989	<.0001	.952	<.0001	<.0001	<.0001	.0023	.038	-
	Emax (%)	Mean	97.54	82.28	95.31	111.9	0.3425	99.92	79.01	81.29	63.32	62.89	ND
		SEM	4.57	4.30	4.11	8.93	1.46	10.97	9.29	12.85	6.62	8.60	ND
		*P* value	-	.127	.788	.28	<.0001	.813	.147	.156	.0008	.005	-
		*N*	8	6	3	3	5	3	6	3	7	3	3
Mm_Fpr1	Log EC50 (M)	Mean	−8.009	−6.291	ND	ND	−5.349	−5.214	−6.155	−6.120	−7.236	−5.849	ND
		SEM	0.187	0.133	ND	ND	0.450	0.358	0.172	0.284	1.957	1.631	ND
		*P* value	-	.0034	-	-	.021	.003	.0013	.001	.714	.259	-
	Emax (%)	Mean	108.1	99.39	ND	ND	92.51	179.4	95.95	66.27	7.393	11.56	ND
		SEM	8.27	6.97	ND	ND	35.00	61.49	8.91	10.48	3.38	12.01	ND
		*P* value	-	.715	-	-	.915	.703	.397	.073	.0008	.005	-
		*N*	3	3	3	3	6	6	5	6	3	3	3
Mm_Fpr2	Log EC50 (M)	Mean	−7.663	−6.956	−6.539	−6.986	−3.797	−4.937	−7.063	−7.106	−8.242	−10.53	−10.52
		SEM	0.082	0.334	0.236	0.350	1.759	0.308	0.436	0.358	0.311	1.846	2.526
		*P* value	-	.038	.0045	.0967	.118	<.0001	.213	.309	.2503	.15	.56
	Emax (%)	Mean	101.7	75.58	118.5	88.01	54.41	81.60	42.90	41.84	43.91	8.683	12.27
		SEM	3.66	11.18	13.24	14.38	138.24	18.54	7.95	5.99	4.85	0.73	1.31
		*P* value	-	.127	.313	.384	.915	.684	.0006	<.0001	<.0001	<.0001	<.0001
		*N*	5	4	3	5	4	5	5	5	6	3	4

*P* values were obtained using multiple *t* tests and indicate a difference from WKYMVm (ND denotes no internalization measured).

WKYMVm produced a clear concentration-dependent increase in internalization at all the receptor subtypes, with a rank order of potency of Hs_FPR2 > Mm_Fpr2 = Hs_FPR1 > Mm_Fpr1 (Supplemental Fig. 7). Hence, all subsequent ligands were normalized to the internalization observed for 1 *μ*M WKYMVm. For Mm_Fpr1, only WKYMVm and its L-enantiomer, WKYMVM, were able to act as full agonists and drive internalization, although WKYMVM was 50-fold less potent at doing so. Of note was some appreciable internalization of Mm_Fpr1 to both bacterial-derived formyl-peptides, fMLF and fMLFK. However, this effect was only apparent at concentrations greater than 1 *μ*M (Fig. 5C). In contrast to Mm_Fpr1, but in line with previous readouts, both fMLF and fMLFK were full agonists at inducing Hs_FPR1 internalization with comparable potencies to WKYMVm (Fig. 5A). Both FPR1 species failed to show appreciable internalization in response to MCT-2, BMS-986235, or MMK1. Finally, the small molecule agonists TCFPR43 and QuinC1 were both partial agonists for all the FPR subtypes tested with both ligands showing the greatest efficacy at Mm_Fpr2.

Comparison between Hs_FPR2 and Mm_Fpr2 revealed a similarity between the rank order of potency between all the ligands tested except for MMK-1, which displayed partial agonism at Hs_FPR2 and was unable to induce internalization of Mm_Fpr2 or indeed any other FPR subtype (Fig. 5B). ACT-389949 is a full agonist at both species of FPR2 and equipotent to WKYMVm at Hs_FPR2, exhibiting 28-fold less potency at Mm_Fpr2. Additionally, ACT-389949 also induces internalization of both the Hs_FPR1 and Mm_Fpr1 subtypes, although only at 10 *μ*M. However, unlike ACT-389949, BMS-986235 was unable to induce any measurable internalization of either species of FPR1 even at the highest concentration tested. In contrast, BMS-986235 induced robust internalization of both FPR2 species and was 9 times more potent at Mm_Fpr2 than Hs_FPR2. Out of all the ligands tested in this study, F2Pal10 was the only ligand unable to induce any appreciable level of internalization at any of the FPRs.

### 3.6 Determination of ligand bias

**Fig. 6:**
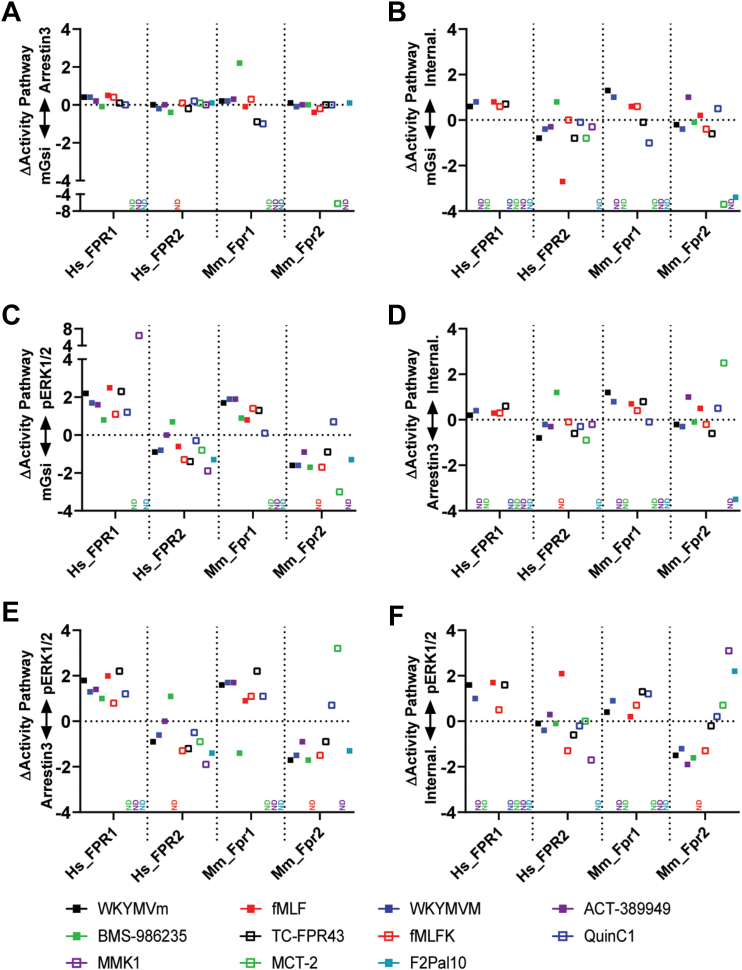
Activity ratio of each receptor and ligand. The activity pathway was determined by subtracting the log (Emax/EC50) of signaling readout 1 from the log (Emax/EC50) of signaling readout 2 to give an indication of bias relative to the position of the reference ligand WKYMVm (black square). ND denotes compounds where no values are available.

It has been widely reported that the FPR family, and FPR2 in particular, display considerable ligand promiscuity to the point whereby the same receptor can mediate proinflammatory or proresolutory responses depending on the ligand.^7^^,^^52^ This phenomenon could be described as a physiological form of ligand bias, and it is reasonable to suggest that signaling bias at the receptor might underlie these observations. To determine ligand bias, a reference ligand is required, that is, “balanced” for the pathways tested.^37^ Our data highlight that the synthetic peptide WKYMVm is a suitable reference ligand, as demonstrated by the comparable efficacies at each of the receptor subtypes exhibited across our assays. We then used measurements of the activity ratio (Log (Emax/EC50)) for each receptor, ligand, and pathway in an attempt to observe any ligand bias (Fig. 6). In most places there was little to no bias observed for any receptor. For example, at mGsi versus Arrestin3 recruitment (Fig. 6A), all ligands (where values were available) show similar Δ Activity pathway values to WKYMVm. Indeed, the only examples where this is not the case are BMS-986235 at Mm_Fpr1 and MCT-2 at Mm_Fpr2; however, both examples here show very low efficacy (<20% of WKYMVm), making interpretation difficult, a known limitation of estimates of bias.^37^ Indeed, this trend was carried over throughout the analysis, where the only evidence of bias was with ligands that are very weak agonists where Emax has not been achieved or partial agonists (also see MMK1 at Hs_FPR1 and fMLF at Hs_FPR2). Moreover, for the majority of each ligand-receptor-transducer pair, there was no obvious bias factor for any ligand with respect to WKYMVm; that is, if a ligand behaved as a full agonist in 1 readout, it behaved as a full agonist within the others. This was also true for partial agonists. Of some note, however, was BMS-986235 at Hs_FPR2, which although approximating a full agonist at each pathway, showed a preference for internalization and pERK1/2 over mGsi and Arrestin 3 recruitment, although this was not recapitulated at the Mm_Fpr2 homologue.

F2Pal10 was an interesting ligand that surprisingly showed little discrimination between Gi and Arrestin 3 recruitment to either human or mouse FPR2 homologues yet was calculated to be biased toward Gi and Arrestin 3 compared to pERK signaling, despite F2Pal10 being a full agonist in pERK1/2 assays and only partial at Gi and Arrestin 3 recruitment, again demonstrating the limitations of this approach. Further calculations of bias regarding internalization were not possible, because no values could be determined; thus, extreme bias is not calculable. Of no doubt, however, was the clear lack of internalization of either FPR2 in response to this pepducin, so it may be considered trafficking biased, although how it induces G-protein activation and ERK signaling remains unclear (Supplemental Fig. 4).

## 4 Discussion

Activation of FPRs is critical for the initiation and regulation of the innate inflammatory response, with FPR1 proposed to have a “proinflammatory” role and FPR2 having a dual role in its ability to seemingly mediate both proinflammatory and proresolution signaling depending on the interacting ligand.^4^^,^^7^^,^^12^ Understanding how a single receptor can interact with a plethora of structurally diverse ligands to initiate opposing downstream effects is critical in determining FPR2 modulation of the inflammatory response and how this may be exploited for therapeutic benefit. Additionally, to aid preclinical development of FPR2-targeted therapies, a better understanding of ligand specificity and efficacy between human and murine FPRs is critical. Furthermore, because many studies have used immune cells as a native expression system, this can lead to potential difficulties in interpreting the data due to the fact that both FPR1 and FPR2 and in some instances, such as monocytes and dendritic cells, FPR3, are also present.^11^ Thus, a clear side-by-side comparison of ligand specificity and efficacy at human and mouse FPRs was needed. Importantly, using a heterologous expression system singly expressing human and mouse FPR1 and FPR2 receptors and comparison, with a “balanced” reference ligand allows, for the first time, several insights into human and mouse FPR pharmacology (summarized in Table 6).

**Table 6:** Summary of main findings

Compound	Selective	Bias	Notes
WKYMVm	−	−	Reference compound
WKYMVM	+ (2>1)	No	
ACT-389949	+ (2>1)	No	
BMS-986235	+++ (2>>1)	+ (Hs_FPR2)	Bias against internalization
fMLF	+++ (1>>2)	No	Weaker at Mm_fpr1
fMLFK	+++ (1>>2)	No	Weaker at Mm_fpr1
TC-FPR43	−	No	Partial/weak only
QuinC1	−	No	Partial/weak only
MCT-2	++ (2>1)	No	Partial agonist
MMK1	+++ (Hs_FPR2)	No	Only activity at Hs_FPR2
F2Pal10	+++ (2>>1)	+++ (Hs_FPR2 and Mm_Fpr2)	Bias against Arr3 and internalization

We have noted that many reportedly FPR subtype-specific ligands are not actually as selective as widely used. For example, WKYMVM is often regarded as being Hs_FPR2 specific after it was found to induce calcium signaling in Hs_FPR2 but not Hs_FPR1 transfected human leukemia (HL60) cells^15^ and has been used in subsequent studies to assess Hs_FPR2 function in polymorphonuclear leukocytes.^53^^,^^54^ Here, we find that WKYMVM is also able to bind both the human and mouse FPR1, induce effective recruitment of mGsi and Arrestin 3, generate a P-ERK response, and drive internalization. However, these responses were typically 100-fold less potent than WKYMVM at the corresponding FPR2 receptor. ACT-389949, 1 of the few ligands to be tested in a clinical trial for FPRs, has also been reported as FPR2 selective.^47^ It was found calcium flux in human neutrophils in response to ACT-389949 was attenuated following pretreatment with an antagonist at FPR2 but not FPR1 (PBP10 or cyclosporin H respectively^55^). Furthermore, arrestin recruitment in response to ACT-389949 using the Pathhunter assay found neither WKYMVM nor ACT-389949 resulted in recruitment to FPR1 (at 100 nM) but did to FPR2 (with an EC50 of 20 nM^55^). Importantly, we find ACT-389949 is able to elicit arrestin and G-protein recruitment and induce robust pERK1/2 at both Hs_FPR1 and Mm_Fpr1, albeit predominantly at concentrations of >100 nM. In extension to the previous findings^47^ we also demonstrate that ACT-389949 is a potent ligand for Mm_Fpr2, with greater efficacy than at Hs_FPR2 across the readouts examined here. Importantly, a second molecule currently being investigated for clinical efficacy is BMS-986235,^56^ which, in contrast to ACT-389949, was evidently more selective for FPR2 over FPR1 in both species, showing little to no efficacy at FPR1, even at 10 *μ*M. It is therefore possible that differences in physiological efficacy might be explained by selective versus dual agonism. It is important to mention that ACT-389949 has been suggested to be unable to initiate a pERK1/2 response,^25^ a result recently disputed.^26^ Our current results agree with the later study as we show that ACT-389949 is able to induce phosphorylation of ERK downstream of all FPRs tested.

The only compound tested that demonstrated clear species and FPR subtype specificity, with measurable effects only at Hs_FPR2, was the peptide MMK1. MMK-1 presented as a partial agonist with respect to the WKYMVm, response except for the p-ERK1/2 response where it behaved as a full agonist but with a 10-fold reduction in potency from WKYMVm. However, this perceived difference in the efficacy of MMK-1 in the different readouts is likely not due to a p-ERK1/2 bias but through signal amplification of downstream signaling pathways versus directly measuring a response at the receptor level (eg, for the G-protein recruitment). The selectivity of MMK-1 for Hs_FPR2 over Hs_FPR1 has been previously demonstrated in HL-60 cells^57^ and in a heterologous HEK293 expression system^18^ where calcium responses were measured. Our findings are at odds with this previous study^18^ as we saw no activity of MMK-1 at Mm_Fpr2; however, we measured phosphorylation of ERK and not calcium signaling, becuase in our hands calcium responses from FPRs (and other Gi-coupled GPCRs) were not detectable. Importantly, mouse neutrophils are not activated by MMK1, consistent with our data here.^58^ While this clear species and FPR2 subtype selectivity might initially appear limiting to the further study of MMK-1 in preclinical models, a human FPR2 knock-in mouse model has recently been developed,^24^ presenting a novel opportunity for the physiological effect of human FPR2 activation to be studied in isolation.

In addition to the discrepancies in FPR subtype selectivity of reported ligands, potential differences in efficacy may also account for the observed physiological differences of FPRs in models of inflammation. For example, the proinflammatory bacterial peptide fMLFK has been proposed to be more potent at Hs_FPR2 than fMLF, which was initially based on characterization in rat basophilic leukemia cells^59^ and molecular docking on more recently acquired cryo-EM structures.^60^ This is due to the overall negative charge of the FPR2 binding pocket and the positive charge of the additional lysine (K) on fMLFK, which directly interacts with asparagine (Asp-281) within the orthosteric binding site of Hs_FPR2, while similar interactions do not take place for fMLF, which binds unfavorably in Hs_FPR2 binding pocket.^59^^,^^60^ In contrast, Hs_FPR1 does not possess an asparagine at this position in its binding pocket but instead possesses tyrosine 257 (Y257), not present in Hs_FPR2, which directly interacts with fMLF and attributes to the potency of fMLF for Hs_FPR1.^60^^,^^61^ Indeed, in line with these previous observations, fMLF was consistently most efficacious at Hs_FPR1 than the other FPR species and subtypes by ∼100-fold. However, in this study, fMLFK does not appear as selective nor as efficacious for Hs_FPR2 and is instead a very weak partial agonist while behaving as a full agonist at Hs_FPR1, showing similar potency and selectivity as fMLF. Moreover, fMLFK and fMLF both behaved as weak partial agonists at Mm_Fpr1, more in line with the responses seen at Hs_FPR2 and Mm_Fpr2 than at Hs_FPR1, which calls into question the functional similarity between human and mouse FPRs as completely interchangeable. In addition, there does appear to be FPR subtype selectivity to longer formylated peptides of mitochondrial origin, MCT-2,^48^ which consistently demonstrated activity, albeit weak in comparison with WKYMVm, at both human and mouse FPR2 over FPR1, indicating that FPR2 may modulate immune responses to damage-associated molecular patterns rather than to bacterial infection.^35^

Ligand bias has been proposed to be of enormous pharmacological value, because biasing signaling toward specific signaling pathways proffers the development of therapeutics with fewer side effects and/or increased ligand efficacy.^22^^,^^23^ Because distinct FPR ligands are reported to cause either proinflammatory responses in the case of formylated bacterial and mitochondrial peptides and serum amyloid A, while others, such as LipoxinA4, AnnexinA1, and ResolvinD1, promote resolution, ligand bias would provide a simple explanation for this phenomenon.^52^^,^^62^ Here, we report that there does not appear to be any obvious ligand-pathway bias that would explain this duality of signaling of the FPR2. Of course, within this study it is not feasible to test all of the reported ligands for FPR2,^28^^,^^29^ nor for all potential G-protein couplings. Further, although the use of overexpression in HEK293 cells is a widely used model for identifying ligand bias, it is possible that the use of an overexpression system has masked evidence of subtle bias,^63^ so the potential of a biased ligand being discovered for either FPR1 or FPR2 remains a formal possibility.

Despite these caveats, of the 11 structurally diverse ligands tested herein, there was only 1 exception that would consistently fulfill the criteria of demonstrating ligand-pathway bias, the previously described pepducin ligand F2Pal10.^39^ This bias effect was seen at both human and mouse FPR2, with no activity observed at Hs_FPR1 or Mm_Fpr1. Pepducins such as F2Pal10 are designed around the peptide sequence of the Hs_FPR2 3rd intracellular loop (ICL) (KIHKKGMIKS), which is supposed to confer subtype specificity.^64^ Critically, there is a greater sequence divergence between Hs_FPR2 3rd ICL and Mm_Fpr2 3rd ICL, which are both activated by F2Pal10, than between Hs_FPR2 and Hs_FPR1 3rd ICL, where F2Pal10 remains inactive.^38^^,^^64^ It therefore remains unclear exactly how F2Pal10 is able to activate these receptors. Here, we show that at higher concentrations F2Pal10 is able to compete with fluorescently labeled WKYMVm for the extracellular orthosteric binding site, suggesting it may not interact with the receptors purely intracellularly. Of further note was the observation that despite clear evidence for inducing heterotrimeric G-protein dissociation and activation of the ERK pathway, with an efficacy similar to WKYMVm, Gi interaction was only detected at 1 and 10 *μ*M, and only to 20% of that seen with WKYMVm. This suggests agonism by F2Pal10 is through a different molecular process, which leaves the receptor unable to interact with arrestins or internalize.

Biased ligands are often reported as preferentially recruiting either G-protein or arrestin to generate downstream signals, typically with p-ERK1/2 used as a readout.^45^^,^^46^ Work by ourselves and others has demonstrated that Hs_FPR2-induced ERK phosphorylation by WKYMVm absolutely requires activation of Gi family G-proteins and was able to occur in the absence of arrestins.^42^, ^43^, ^44^ It is therefore unclear what beneficial signaling outcome would result from an arrestin-biased ligand; however, the extent of arrestin recruitment induced by each ligand may have an important role to play in regulating ligand tolerance at Hs_FPR2. Recently, it has been proposed that the potency of arrestin recruitment observed with ACT-389949 compared to BMS-986235 was responsible for internalization and apparent downregulation of FPR2 following ACT-389949 that halted clinical development of this ligand and why BMS-986235 may be a superior compound.^24^^,^^50^ This hypothesis is based on the model of arrestin association determining receptor postendocytic fate,^65^ because the postendocytic recycling of FPR2 was greater for BMS-986235-treated cells than those treated with ACT-389949.^24^ Here, we observed near-identical potency of arrestin recruitment to Hs_FPR2 following ACT-389949 activation (EC50 of 18 nM) using our BRET assay as has been previously reported as measured by the Pathhunter assay.^55^ In contrast, previous assessments of arrestin recruitment to Hs_FPR2 following BMS-986235 by both the Pathhunter (EC50 130 nM)^66^ and BRET (EC50 250 nM) assay^56^ were greater than the EC50 we observed herein (39.9 nM, Table 4). This discrepancy in the relative potency of BMS-986235 may be due to the difference in the acceptor-donor system used. Here, we used the Renilla Luciferase (Rluc8) directly fused to the C-terminal of Hs_FPR2 as a BRET donor, with Arrestin 3-YFP as the acceptor. This contrasts with the arrestin-luciferase donor and membrane-bound acceptor (green fluorescent protein fused to a C-A-A-X motif) used previously, a system that indirectly measures arrestin interaction with the receptor (bystander BRET^56^). Interestingly, we observe a slight bias away from internalization for BMS-986235, consistent with recent publications, suggesting that it is less potent at driving endocytic trafficking^24^; however, this is only observed at the Hs_FPR2, with no bias seen at Mm_Fpr2. Further, we do not see similar bias against Arrestin 3 recruitment in response to BMS-986235, consistent with our previous observation that Arrestin recruitment and Hs_FPR2 internalization are not necessarily causally linked.^44^ Overall, however, our data support the finding that ACT-389949 is more potent at inducing internalization of Hs_FPR2 than BMS-986235.^24^^,^^26^ Whether these differences result in altered postendocytic trafficking and downregulation of the Hs_FPR2 remains to be confirmed; however, it is important to note that our previous studies indicate that the association of Hs_FPR2 with arrestins does not absolutely determine the postendocytic fate of FPR2 following WKYMVm activation.^30^^,^^44^ Thus far, the role of arrestin in regulating FPR postendocytic fate in response to other ligands has not yet been addressed but is clearly an important aspect to in the consideration of determining the long-term responsiveness of FPRs to treatment.

Finally, for a receptor to induce such a diverse array of signaling outcomes, it could be expected that the FPR2 was able to adopt several active conformations, because is proposed to explain ligand bias at several GPCRs.^67^ So far there has only been 1 crystal structure resolved for the FPR2, with WKYMVm occupying the orthosteric binding pocket^60^ and few cryo-EM structures of FPR2 and FPR1.^68^^,^^69^ These have revealed FPR2 to possess a much wider orthosteric binding pocket than FPR1, which may explain how larger ligands can supposedly bind to FPR2. Importantly, overlaying of cryo-EM structures of FPR2 coupled to Gi for WKYMVm, TCFPR43 (Cp43), and fMLFKII there was great similarity in the structures that would not account for the large physiological differences observed for these ligands.^69^ There is currently no structural data available for any of the mouse FPRs, highlighting that further expansion of structural data is required, particularly comparison of the receptors in an inactive or antagonist-bound state or with arrestin to aid in structure-based drug design and identification of potent and selective therapeutics for these receptors.

Overall, from the results herein and conclusions available from current FPR structural data, we tentatively suggest that it is unlikely that proposed proinflammatory or proresolution ligands cause unique acute signaling signatures at FPR2 and that the described physiological difference of these ligands observed both in isolated cells and in vivo murine models is likely more readily explained by previously unappreciated low FPR subtype specificity and differences in ligand efficacy, pharmacokinetics, or stability.

## Conflict of interest

The authors declare no conflicts of interest.
