# Supplementary material for: Investigation into ligand selectivity and bias at the formyl peptide receptor family
Source: J Pharmacol Exp Ther. 2025 Oct 28;393(1):103764. doi: 10.1016/j.jpet.2025.103764 (PMC12881670; doi:10.1016/j.jpet.2025.103764)
Supplement: Supplementary Material [file mmc1.pptx]

## Slide 1
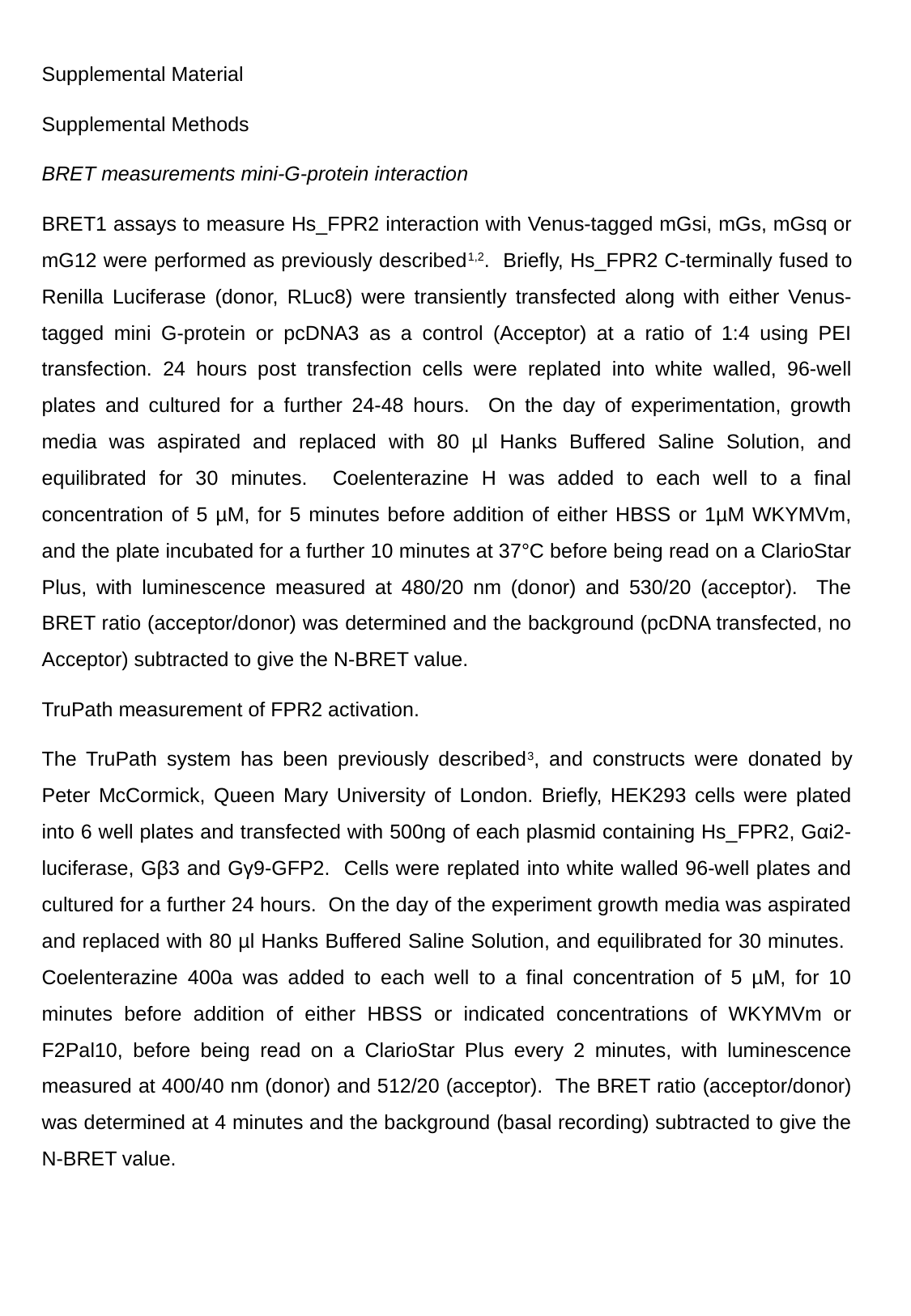

Supplemental Material
Supplemental Methods
BRET measurements mini-G-protein interaction
BRET1 assays to measure Hs_FPR2 interaction with Venus-tagged mGsi, mGs, mGsq or mG12 were performed as previously described1,2. Briefly, Hs_FPR2 C-terminally fused to Renilla Luciferase (donor, RLuc8) were transiently transfected along with either Venus-tagged mini G-protein or pcDNA3 as a control (Acceptor) at a ratio of 1:4 using PEI transfection. 24 hours post transfection cells were replated into white walled, 96-well plates and cultured for a further 24-48 hours. On the day of experimentation, growth media was aspirated and replaced with 80 µl Hanks Buffered Saline Solution, and equilibrated for 30 minutes. Coelenterazine H was added to each well to a final concentration of 5 µM, for 5 minutes before addition of either HBSS or 1µM WKYMVm, and the plate incubated for a further 10 minutes at 37°C before being read on a ClarioStar Plus, with luminescence measured at 480/20 nm (donor) and 530/20 (acceptor). The BRET ratio (acceptor/donor) was determined and the background (pcDNA transfected, no Acceptor) subtracted to give the N-BRET value.
TruPath measurement of FPR2 activation.
The TruPath system has been previously described3, and constructs were donated by Peter McCormick, Queen Mary University of London. Briefly, HEK293 cells were plated into 6 well plates and transfected with 500ng of each plasmid containing Hs_FPR2, Gαi2-luciferase, Gβ3 and Gγ9-GFP2. Cells were replated into white walled 96-well plates and cultured for a further 24 hours. On the day of the experiment growth media was aspirated and replaced with 80 µl Hanks Buffered Saline Solution, and equilibrated for 30 minutes. Coelenterazine 400a was added to each well to a final concentration of 5 µM, for 10 minutes before addition of either HBSS or indicated concentrations of WKYMVm or F2Pal10, before being read on a ClarioStar Plus every 2 minutes, with luminescence measured at 400/40 nm (donor) and 512/20 (acceptor). The BRET ratio (acceptor/donor) was determined at 4 minutes and the background (basal recording) subtracted to give the N-BRET value.

## Slide 2
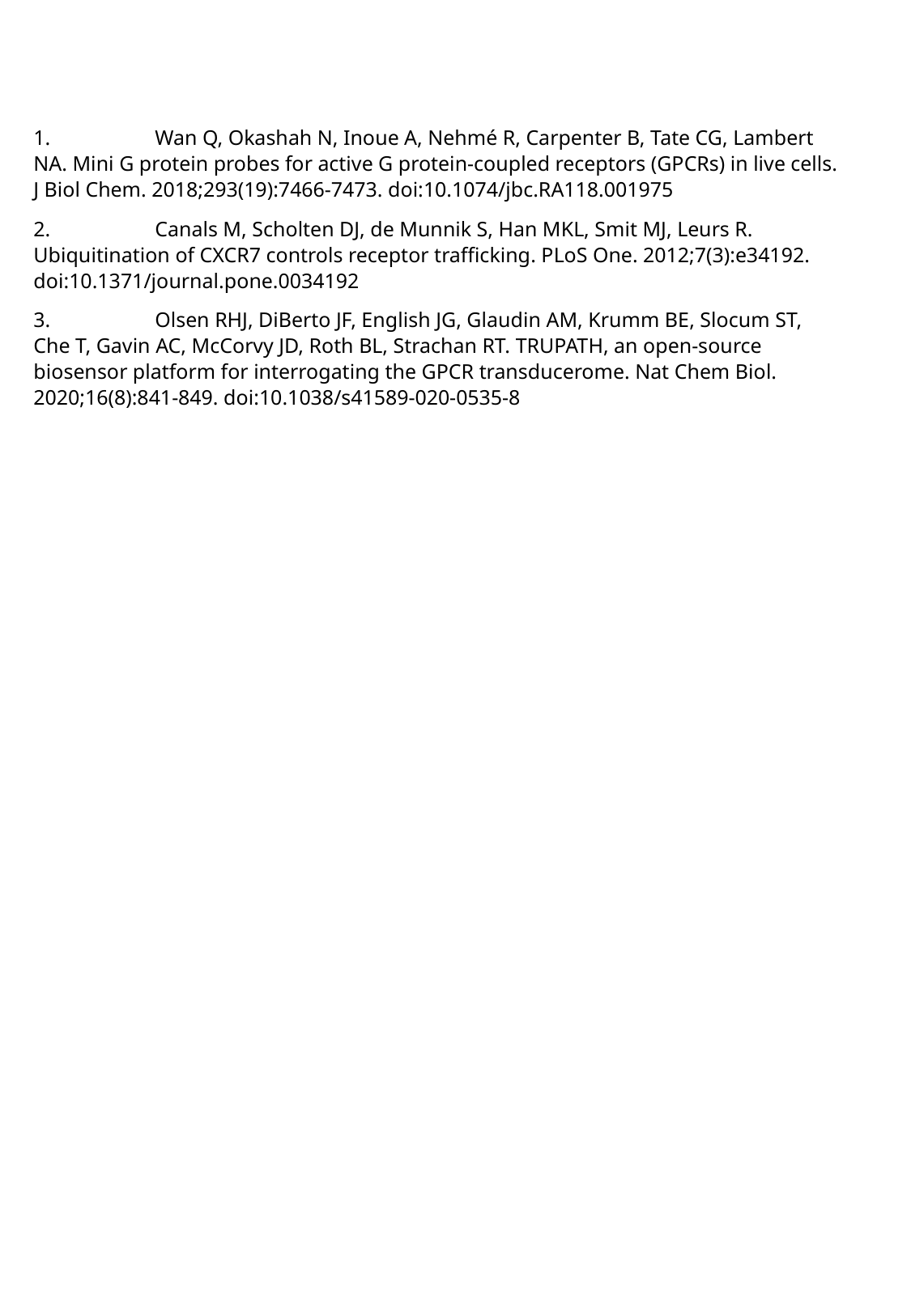

1. 	Wan Q, Okashah N, Inoue A, Nehmé R, Carpenter B, Tate CG, Lambert NA. Mini G protein probes for active G protein-coupled receptors (GPCRs) in live cells. J Biol Chem. 2018;293(19):7466-7473. doi:10.1074/jbc.RA118.001975
2. 	Canals M, Scholten DJ, de Munnik S, Han MKL, Smit MJ, Leurs R. Ubiquitination of CXCR7 controls receptor trafficking. PLoS One. 2012;7(3):e34192. doi:10.1371/journal.pone.0034192
3. 	Olsen RHJ, DiBerto JF, English JG, Glaudin AM, Krumm BE, Slocum ST, Che T, Gavin AC, McCorvy JD, Roth BL, Strachan RT. TRUPATH, an open-source biosensor platform for interrogating the GPCR transducerome. Nat Chem Biol. 2020;16(8):841-849. doi:10.1038/s41589-020-0535-8

## Slide 3
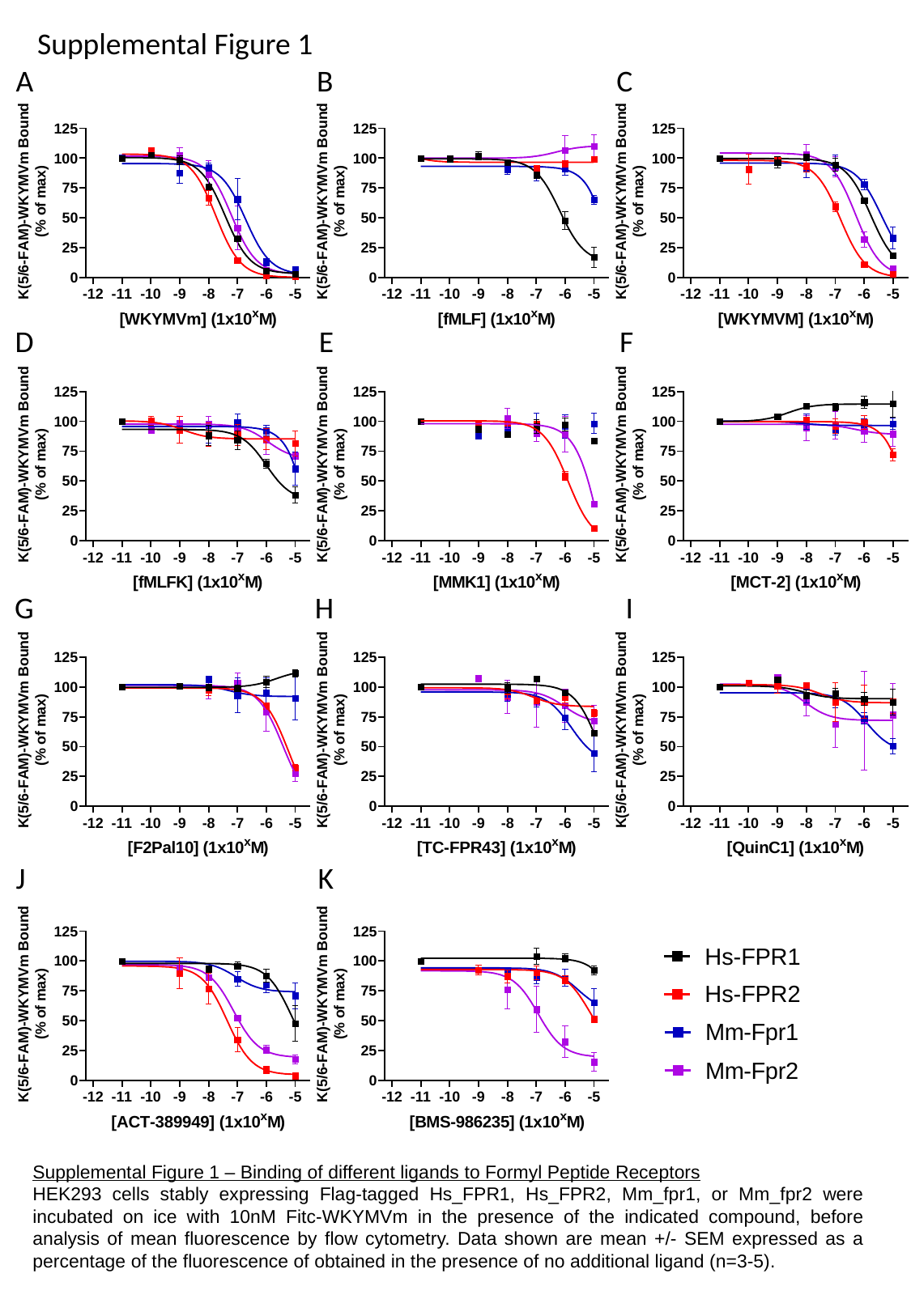

Supplemental Figure 1
A
B
C
D
E
F
G
H
I
J
K
Supplemental Figure 1 – Binding of different ligands to Formyl Peptide Receptors
HEK293 cells stably expressing Flag-tagged Hs_FPR1, Hs_FPR2, Mm_fpr1, or Mm_fpr2 were incubated on ice with 10nM Fitc-WKYMVm in the presence of the indicated compound, before analysis of mean fluorescence by flow cytometry. Data shown are mean +/- SEM expressed as a percentage of the fluorescence of obtained in the presence of no additional ligand (n=3-5).

## Slide 4
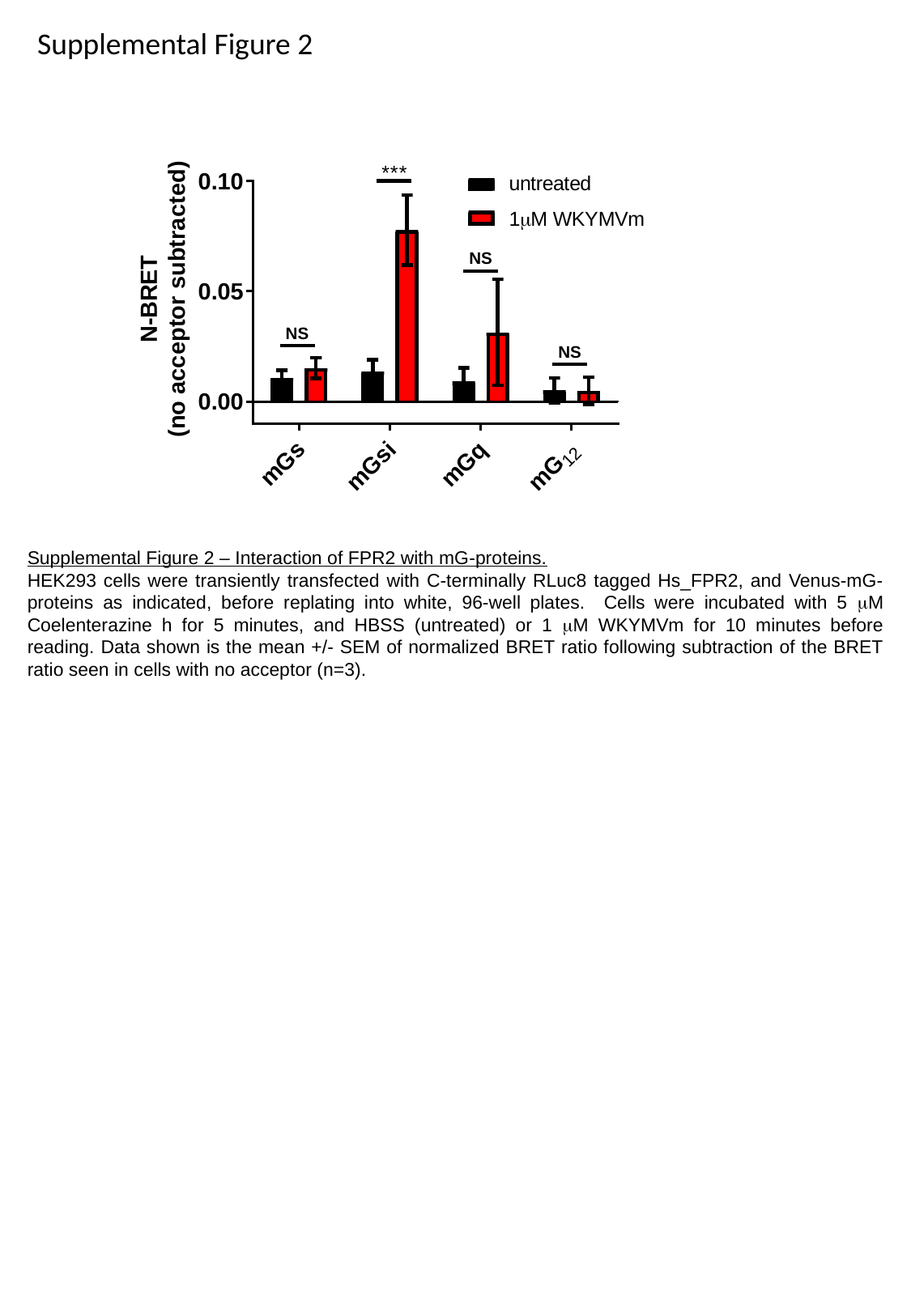

Supplemental Figure 2
Supplemental Figure 2 – Interaction of FPR2 with mG-proteins.
HEK293 cells were transiently transfected with C-terminally RLuc8 tagged Hs_FPR2, and Venus-mG-proteins as indicated, before replating into white, 96-well plates. Cells were incubated with 5 mM Coelenterazine h for 5 minutes, and HBSS (untreated) or 1 mM WKYMVm for 10 minutes before reading. Data shown is the mean +/- SEM of normalized BRET ratio following subtraction of the BRET ratio seen in cells with no acceptor (n=3).

## Slide 5
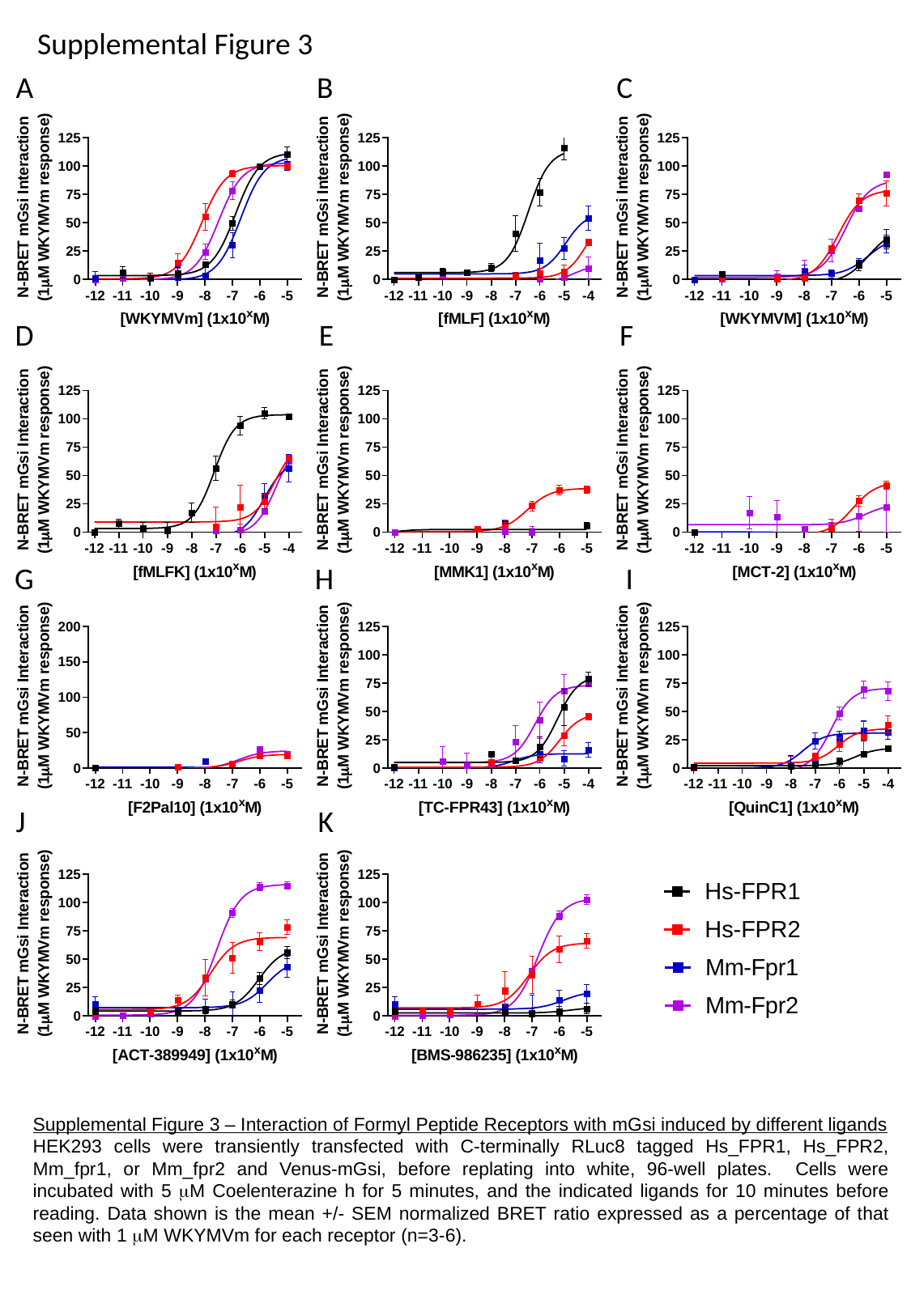

Supplemental Figure 3
A
B
C
D
E
F
G
H
I
J
K
Supplemental Figure 3 – Interaction of Formyl Peptide Receptors with mGsi induced by different ligands
HEK293 cells were transiently transfected with C-terminally RLuc8 tagged Hs_FPR1, Hs_FPR2, Mm_fpr1, or Mm_fpr2 and Venus-mGsi, before replating into white, 96-well plates. Cells were incubated with 5 mM Coelenterazine h for 5 minutes, and the indicated ligands for 10 minutes before reading. Data shown is the mean +/- SEM normalized BRET ratio expressed as a percentage of that seen with 1 mM WKYMVm for each receptor (n=3-6).

## Slide 6
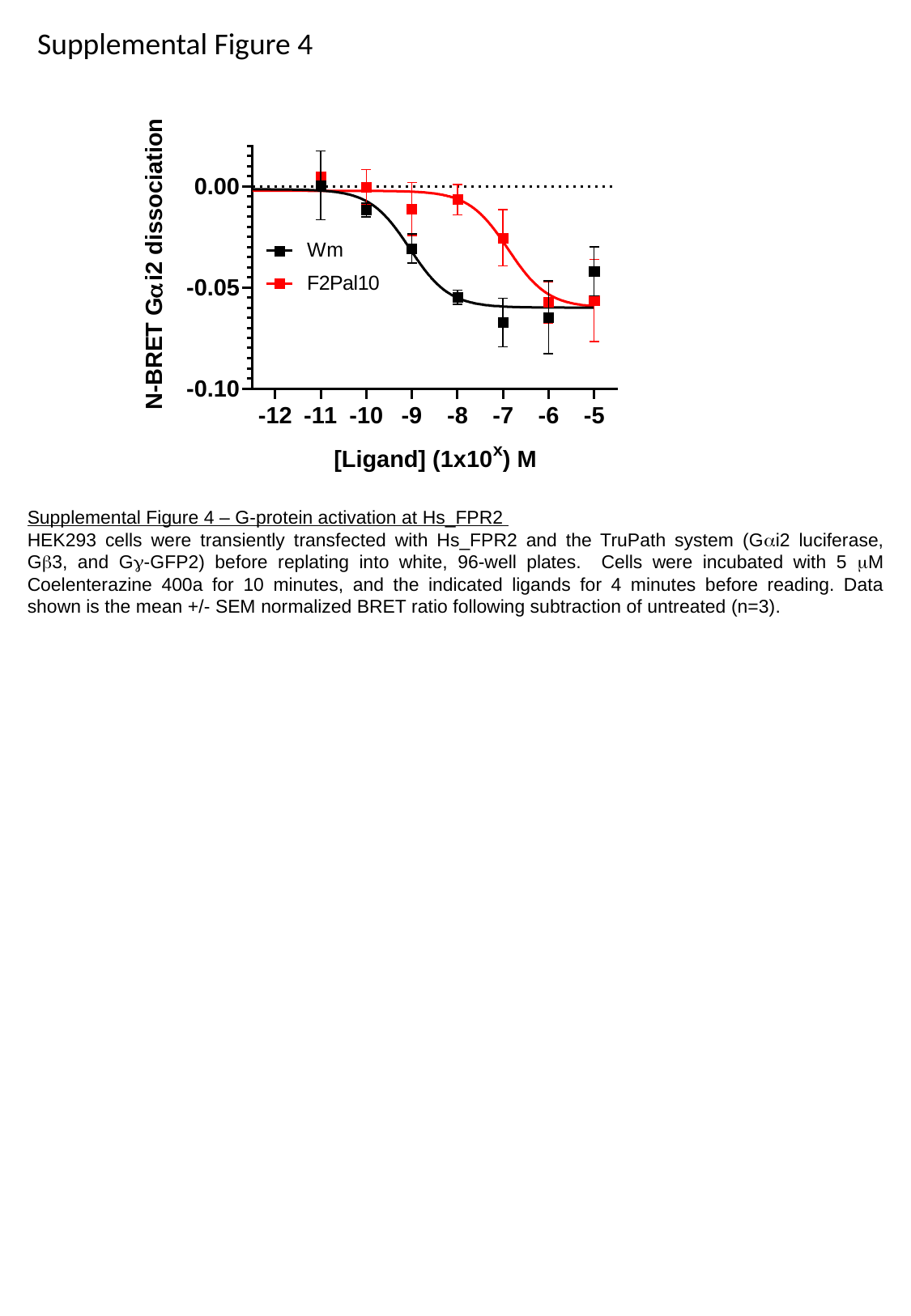

Supplemental Figure 4
Supplemental Figure 4 – G-protein activation at Hs_FPR2
HEK293 cells were transiently transfected with Hs_FPR2 and the TruPath system (Gai2 luciferase, Gb3, and Gg-GFP2) before replating into white, 96-well plates. Cells were incubated with 5 mM Coelenterazine 400a for 10 minutes, and the indicated ligands for 4 minutes before reading. Data shown is the mean +/- SEM normalized BRET ratio following subtraction of untreated (n=3).

## Slide 7
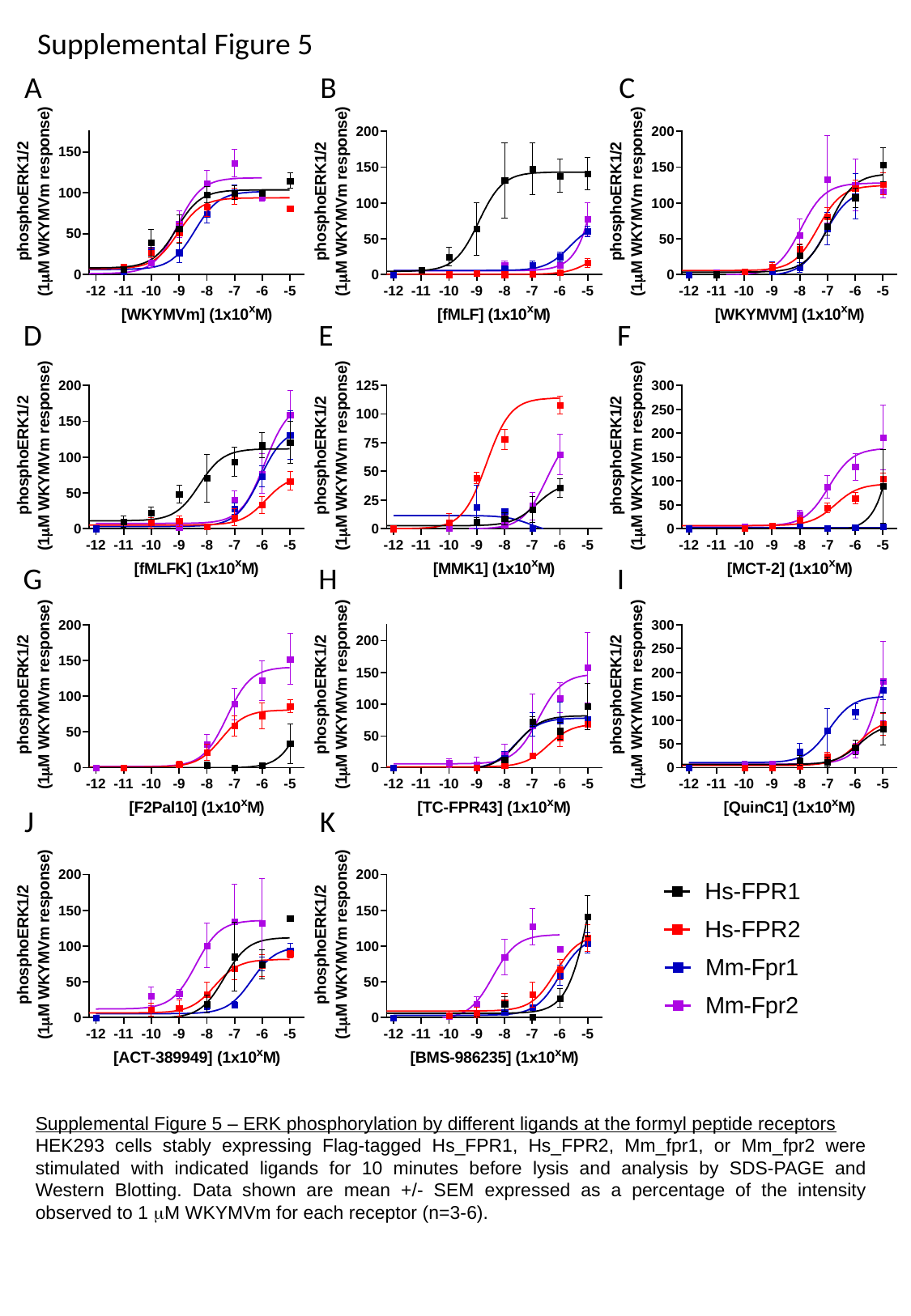

Supplemental Figure 5
A
B
C
D
E
F
G
H
I
J
K
Supplemental Figure 5 – ERK phosphorylation by different ligands at the formyl peptide receptors
HEK293 cells stably expressing Flag-tagged Hs_FPR1, Hs_FPR2, Mm_fpr1, or Mm_fpr2 were stimulated with indicated ligands for 10 minutes before lysis and analysis by SDS-PAGE and Western Blotting. Data shown are mean +/- SEM expressed as a percentage of the intensity observed to 1 mM WKYMVm for each receptor (n=3-6).

## Slide 8
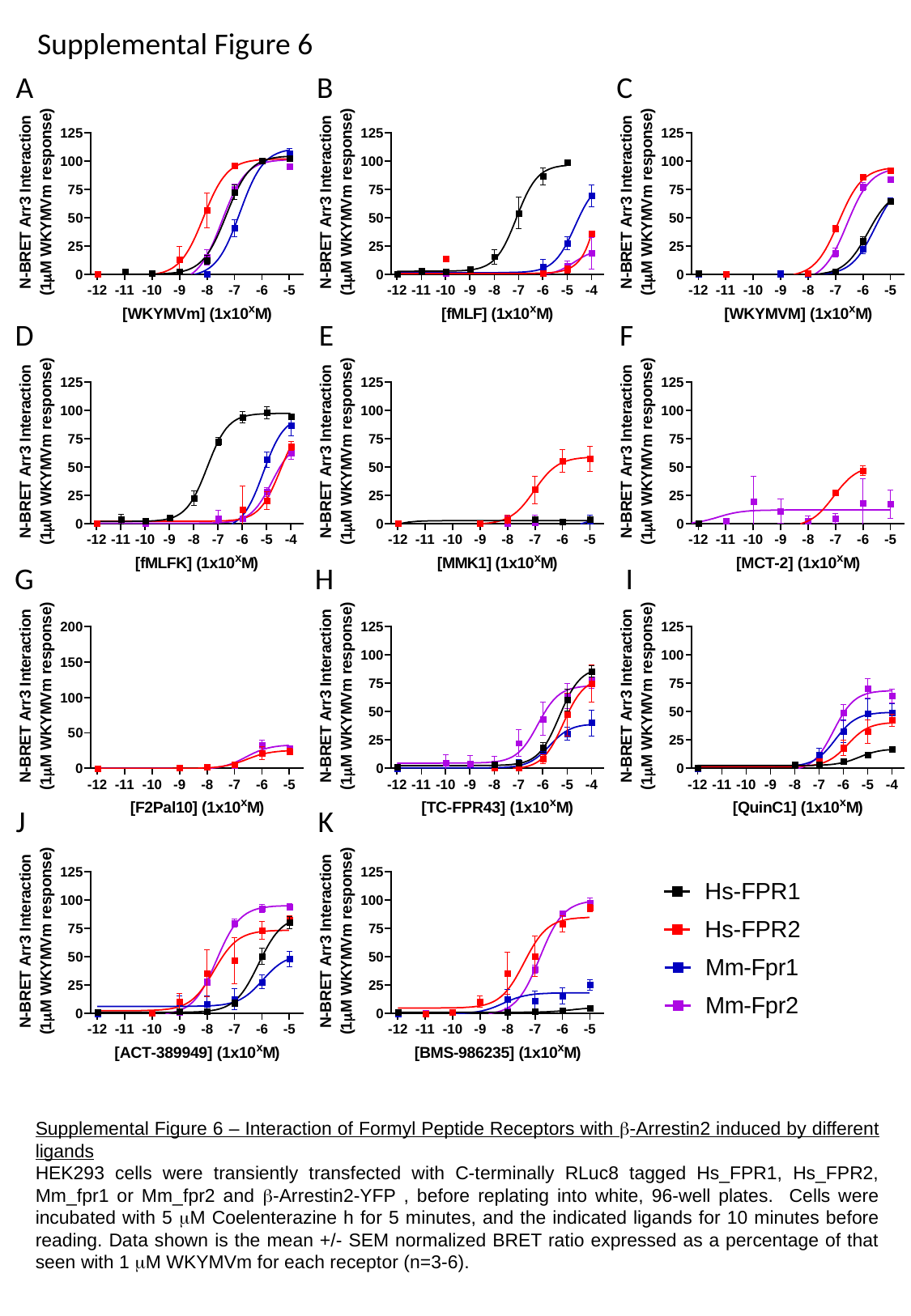

Supplemental Figure 6
A
B
C
D
E
F
G
H
I
J
K
Supplemental Figure 6 – Interaction of Formyl Peptide Receptors with b-Arrestin2 induced by different ligands
HEK293 cells were transiently transfected with C-terminally RLuc8 tagged Hs_FPR1, Hs_FPR2, Mm_fpr1 or Mm_fpr2 and b-Arrestin2-YFP , before replating into white, 96-well plates. Cells were incubated with 5 mM Coelenterazine h for 5 minutes, and the indicated ligands for 10 minutes before reading. Data shown is the mean +/- SEM normalized BRET ratio expressed as a percentage of that seen with 1 mM WKYMVm for each receptor (n=3-6).

## Slide 9
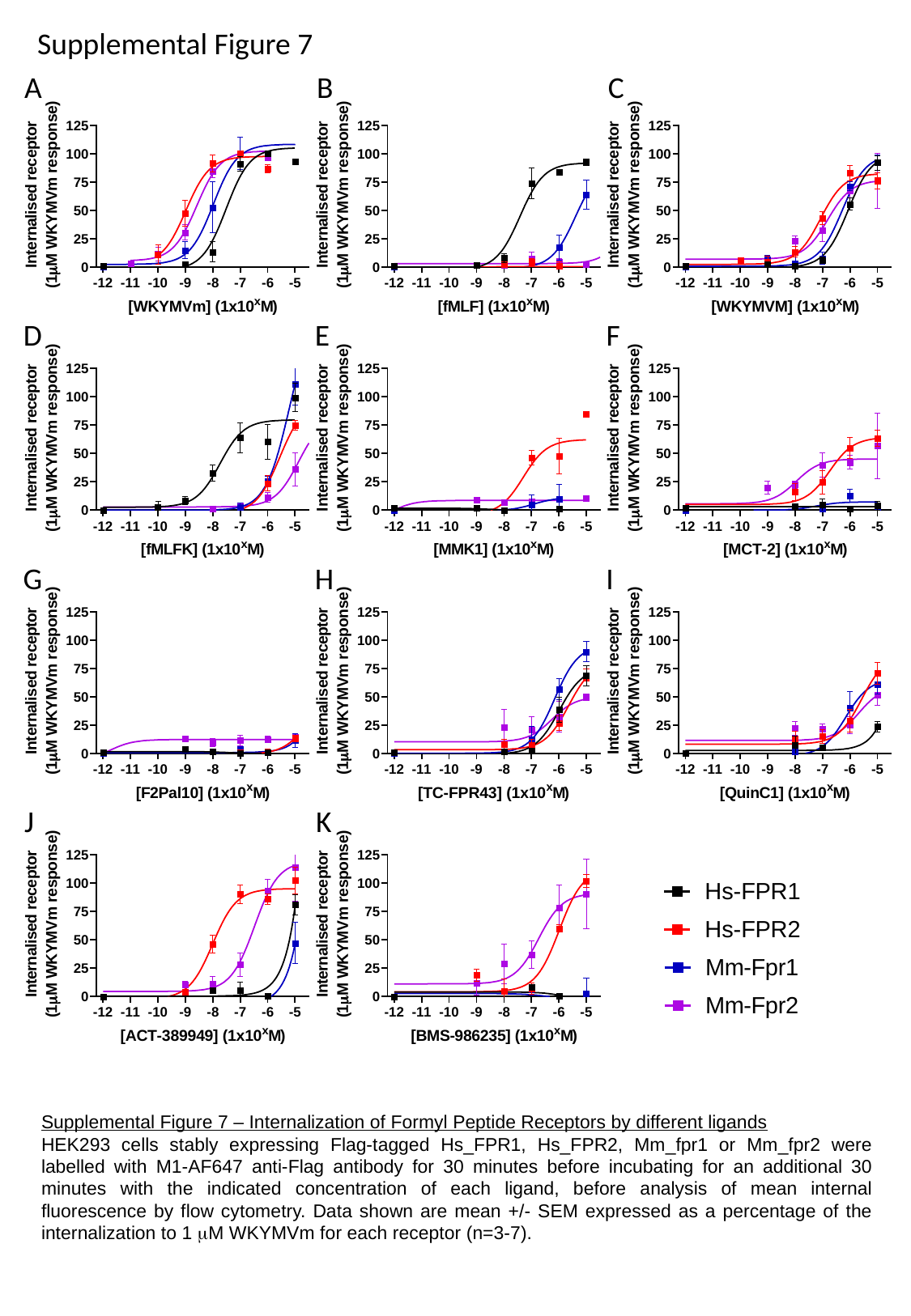

Supplemental Figure 7
A
B
C
D
E
F
G
H
I
J
K
Supplemental Figure 7 – Internalization of Formyl Peptide Receptors by different ligands
HEK293 cells stably expressing Flag-tagged Hs_FPR1, Hs_FPR2, Mm_fpr1 or Mm_fpr2 were labelled with M1-AF647 anti-Flag antibody for 30 minutes before incubating for an additional 30 minutes with the indicated concentration of each ligand, before analysis of mean internal fluorescence by flow cytometry. Data shown are mean +/- SEM expressed as a percentage of the internalization to 1 mM WKYMVm for each receptor (n=3-7).
